# Evaluating the health risk of probiotic supplements from the perspective of antimicrobial resistance

**DOI:** 10.1128/spectrum.00019-24

**Published:** 2024-12-10

**Authors:** Qiwen Tian, Hailv Ye, Xuan Zhou, Junyi Wang, Lifang Zhang, Wenxuan Sun, Chenxin Duan, Minyu Fan, Wei Zhou, Chuyun Bi, Qiong Ye, Aloysius Wong

**Affiliations:** 1 Department of Biology, College of Science, Mathematics and Technology, Wenzhou-Kean University, Wenzhou, Zhejiang, China; 2 Department of Biology, Dorothy and George Hennings College of Science, Mathematics and Technology, Kean University, Union, New Jersey, USA; 3 Zhejiang Bioinformatics International Science and Technology Cooperation Center, Wenzhou, Zhejiang, China; 4 Wenzhou Municipal Key Lab for Applied Biomedical and Biopharmaceutical Informatics, Wenzhou, Zhejiang, China; University of Maryland Eastern Shore, Princess Anne, Maryland, USA

**Keywords:** probiotics, antimicrobial resistance, probiotic supplements, horizontal gene transfer

## Abstract

Antimicrobial resistance remains a public health threat. Probiotics harboring antimicrobial resistant genes (ARGs) have, in recent years, been considered a potential health risk. Studies conducted on probiotics from increasingly popular health supplements have raised the possibility of transmitting ARGs to commensals in the human gut, concomitantly establishing a reservoir of ARGs and risking acquisition by opportunistic pathogens. Building on our previous study that reported multiple antibiotic resistance in probiotics of health supplements, in this research, we have attempted to detect their ARGs that may account for resistant phenotypes. ARGs responsible for tetracycline, macrolide, aminoglycoside, and glycopeptide resistance were prevalent in probiotics. Through laboratory adaptive evolution studies, we also show that streptomycin-adapted probiotics gained resistance to erythromycin, tetracycline, and doxycycline more effectively than non-adapted ones. When co-incubated with *Enterococcus faecalis*, *Escherichia coli*, or *Staphylococcus aureus* on Caco-2 and/or HCT-116 cells, streptomycin resistance was transferred from the adapted probiotics to generate transconjugants at frequencies comparable to or higher than that of other studies conducted through filter mating. Consistently, ARGs conferring resistance to streptomycin (*aadA*) and erythromycin [*erm(B)−1*] were detected in *E. coli* and *S. aureus* transconjugants, respectively, after co-incubation with streptomycin-adapted probiotics on Caco-2 cells. *aadA* and *erm(B)−1* were both detected in *E. faecalis* transconjugant after the same co-incubation on HCT-116 cells. Our data and future comparative genomics and metagenomics studies conducted on animal models and in healthy, immunocompromised, and/or antibiotic-treated human cohorts will contribute to a more comprehensive understanding of probiotic consumption, application, and safety.

**IMPORTANCE:**

Probiotics are becoming increasingly popular, with promising applications in food and medicine, but the risk of transferring ARGs to disease-causing bacteria has raised concerns. Our study detected ARGs in probiotics of health supplements conferring resistance to tetracycline, macrolide, aminoglycoside, and glycopeptide drugs. Streptomycin-adapted probiotics also gained resistance to other antibiotics more effectively than non-adapted ones. Importantly, we showed that streptomycin resistance could be transferred to other bacteria after co-incubation with probiotics on human intestinal cells. ARGs responsible for erythromycin and streptomycin resistance, which were initially absent in the recipient bacteria, were also detected in the transconjugants. Our data build the foundation for future studies that will be conducted on animal models and in humans and leveraging advanced metagenomics approaches to clarify the long-term health risk of probiotic consumption.

## INTRODUCTION

**Fig 1:**
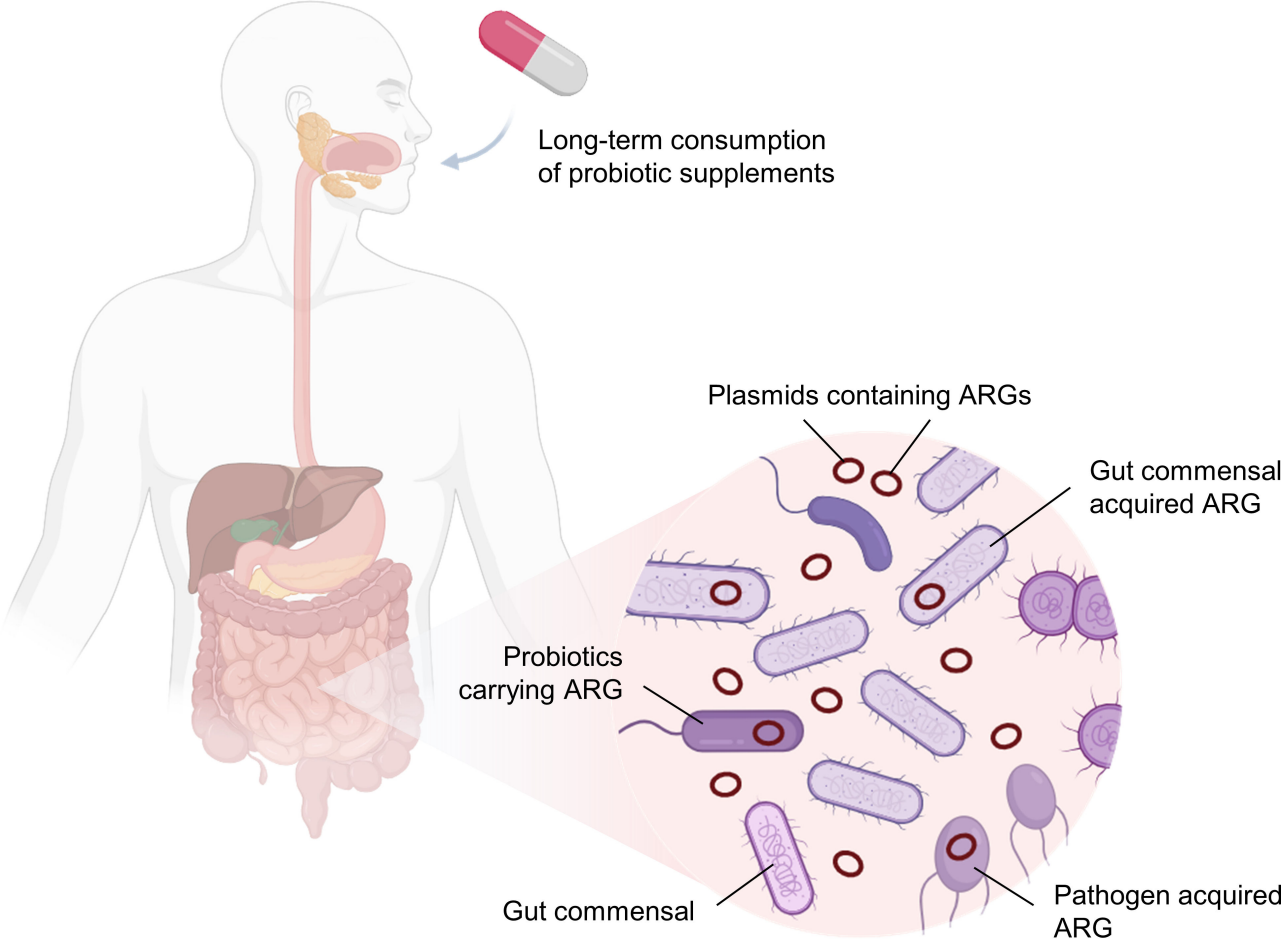
A model illustrating the plausible transfer of antimicrobial resistance genes (ARGs) (red circles) from probiotics of health supplements to commensals and/or pathogens in the human gut. Created with BioRender.com.

Bacterial antimicrobial resistance (AMR) has been linked to approximately 5 million deaths in 2019 (1). This impact was further exacerbated during the severe acute respiratory syndrome coronavirus 2 pandemic as antimicrobial drugs were used much more frequently to treat secondary infections (2, 3). The World Health Organization has consistently classified AMR as one of the top 10 global public health threats (4, 5). Probiotics, which are known to confer many health benefits, such as the alleviation of lactose intolerance, the reduction of fecal enzymes and mutagenicity, the lowering of cholesterol level, and the prevention of gastrointestinal diseases (6–8), have emerged as one potential risk for AMR transmission (9–12). Although probiotics are increasingly incorporated into various foods and therapeutic methods, recent studies have raised several detrimental health effects associated with probiotics, including identifying them as potential donors of antimicrobial resistance genes (ARGs) (13–15). The long-term consumption of probiotics, especially in high doses, in the form of health or dietary supplements (14–16) could elevate the rate of ARG transmission from probiotics to commensal bacteria, concomitantly establishing a reservoir of ARGs in the gut and posing a clinical risk when the ARGs are acquired by opportunistic pathogens (Fig. 1) (17–21).

One recent research has directly linked the use of probiotics in humans to the expansion of the gut resistome (19, 20), while isolated reports have previously hinted that ARGs could be transmitted from probiotics to other bacteria, including pathogens, through *in vitro* filter mating approaches (22–25). Studies that focus on the transfer of ARGs examined on surfaces more closely resembling that of the human gut would offer a better understanding of the risk of consuming probiotics harboring ARGs (26). As such, the use of intestinal cell lines, such as the human colorectal adenocarcinoma cells (Caco-2) and the human colorectal carcinoma cells (HCT-116), as models to examine the transmission of ARGs from probiotics to representative bacteria could be more revealing (27, 28). Caco-2, for instance, are highly differentiated cells capable of forming tight junctions and microvilli, producing brush border enzymes, and serving as a popular model in biomedical research for the study of drug transport, absorption, and permeability, as well as microbial interaction (29–32).

Our previous study has shown that probiotics from health supplements could adapt up to 512 µg/mL streptomycin (15), an antimicrobial drug that is used clinically to treat a variety of bacterial infections, including Mycobacterium tuberculosis (33). Another study also showed that the probiotic strain *Lactobacillus plantarum* ATCC14917 could adapt to 2,048 µg/mL streptomycin (34). As a broad-spectrum antibiotic, streptomycin is effective against most Gram-negative and several Gram-positive bacteria, for example, in combination with other antibiotics, when treating *Enterococcus faecalis* and *Escherichia coli* infections (33). Streptomycin interrupts bacterial protein synthesis by targeting the 30S ribosomal subunit, and the binding of which interferes with the correct alignment of tRNA and the mRNA codons leading to the production of faulty proteins (35). Therefore, in this study, we examined the transmission of streptomycin resistance from the probiotics, which we have previously adapted to high dosage of streptomycin to representative bacteria (15), on human intestinal cells. In addition to the determination of transconjugant frequencies, we also detected the ARGs that might be responsible for conferring streptomycin resistance in the representative recipient bacteria.

Multiple studies have also shown that probiotics could tolerate a broad range of antibiotics, and prolonged exposure to the same antibiotics, especially in gradual dosage increments, could encourage the accumulation of beneficial mutations that afford probiotics tolerance to high doses of antibiotics (12, 13, 15). For instance, previous laboratory evolution studies have revealed that *Lactobacillus plantarum* P-8 could adapt up to 16 µg/mL ampicillin (36), while *L. casei* Zhang could adapt up to 8 µg/mL amoxicillin and 32 µg/mL gentamycin (37). It is also conceivable that probiotics already adapted to one antibiotic could adapt more effectively to tolerate other antibiotics, especially if they are of the same class and have a similar mechanism of actions (38). Since the prescription of probiotics during or after a course of antibiotics is a common clinical practice (39, 40), in this study, we also examined the rate at which streptomycin-adapted probiotics evolve to become resistant to other antibiotics through gradual laboratory evolution studies and compared that with non-adapted probiotics.

## RESULTS

### ARGs detected in probiotics from health supplements

Commercially available probiotic supplements obtained in our previous study (15) and their bacteria composition are listed in Table 1. Only *Lactobacillus* probiotic strains were considered in our study as the de Man, Rogosa, and Sharpe (MRS) culture media and our aerobic culture condition preferentially favor the growth of *Lactobacillus*. Other probiotic strains, such as *Enterococcus* and *Streptococcus*, could not grow on this medium or would require a strict anaerobic condition as in the case of *Bifidobacteria* (41–43). Bacteria viability was confirmed in our previous study, and the estimation of viable *Lactobacillus* bacteria amounts recovered from each product has been performed previously using the drop plate method. Except for product F, the enumerated amounts were all close to those listed on the product labels (15).

**TABLE 1:** PCR detection of ARGs in in the DNA extracted from probiotics of health supplements

Product	A	B	C	D	E	F
Probiotic bacteria	*Lactobacillus casei* *Bifidobacterium breve* *Bifidobacterium longum* *Lactobacillus rhamnosus* *Lactobacillus acidophilus* *Lactobacillus plantarum* *Lactobacillus helveticus*	*Lactobacillus acidophilus* *Bifidobacterium animalis* *Lactobacillus plantarum* *Lactobacillus rhamnosus* *Lactobacillus gasseri* *Lactobacillus casei*	*Lactobacillus acidophilus* *Bifidobacterium bifidum* *Bifidobacterium longum* *Lactobacillus casei* *Lactobacillus rhamnosus* *Lactobacillus salivarius*	*Bifidobacterium* *Lactobacillus* *Enterococcus*	*Bifidobacterium* *Lactobacillus* *Streptococcus*	*Lactobacillus reuteri*
ARGs	*aph(3″)-III, tet(M), tet(W), bla, rpoB, msrA*	*aac(6′)-aph(2″), strA, strB, aadA tet(K), tet(W), tet(O), erm(B)-1, bla, vanE, msrA/B, msrA*	*tet(M), tet(K), tet(W), Tn554*	*tet(K), tet(O), bla*	*aac(6′)-aph(2″), aph(3″)-III, tet(K), vanE, vatE, rpoB, parC, msrA*	*tet(K), cat, vatC, vatE, rpoB, parC*

In this study, we attempted to detect the ARGs in the DNA extracted from the probiotics of all the health supplements through PCR using gene-specific primers gathered from the literature and those that were also used in similar studies that examined the safety of probiotics, including antimicrobial resistance assessment (Table S1) (14). We found tetracycline resistance genes in all products, with *tet(K*) being the most common. ARGs responsible for resistance to macrolides (*msrA* and *msrA/B*) and ampicillin (*bla*) were also prevalent in the probiotic supplements. Our previous study showed that probiotics from product B were phenotypically resistant to the greatest number of antibiotics, including teicoplanin, vancomycin, amikacin, tobramycin, ciprofloxacin, and sparfloxacin, as determined through the disc-diffusion method (15). Consistently, in the current study, we detected a total of 12 ARGs that are known to confer resistances to tetracycline, macrolide, aminoglycoside, and glycopeptide antibiotics in probiotics of product B. In comparison, there were eight or fewer ARGs detected in probiotics of other products (Table 1). Notably, ARGs conferring resistance to streptomycin *strA*, *strB*, and *aadA* were detected only in probiotics from product B.

### B^strR^ adapted more efficiently to antibiotics than the non-adapted probiotics

**Fig 2:**
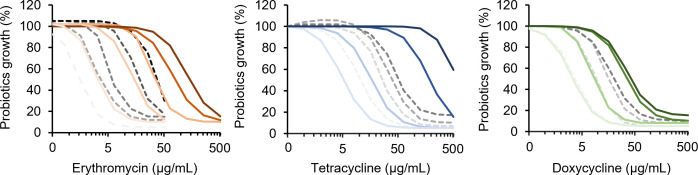
Adaptive evolution of probiotics to antibiotics. The non-adapted and streptomycin-adapted probiotics B^WT^ and B^strR^ are represented as dotted and solid lines, respectively. Line color intensities represent increasing number of adaptive evolution cycle. Dose–response curves were generated from data points of four biological replicates for each antibiotic concentration. Raw data of each cycle represented in this figure, averages, standard deviation, and standard error of the mean are shown in Table S2 and colored corresponding to the line colors in this figure.

In our previous study, probiotics of product B have been adapted up to 512 µg/mL streptomycin (B^strR^) (15). Here, we conducted the same laboratory evolution as in our previous study that was also adopted in similar studies examining antimicrobial resistance in *E. coli* (15, 44) and found that B^strR^ could adapt to erythromycin, tetracycline, and doxycycline more effectively than the non-adapted ones (B^WT^) (Fig. 2; Table S2). Probiotics are assigned ‘resistance’ if they exceed the microbiological breakpoints for *Lactobacillus* strains listed in the guideline provided by the European Food Safety Authority (EFSA) and ‘susceptible’ if they fall below the EFSA breakpoints (45). According to the EFSA, the highest cut-off values for *Lactobacillus* are 64 µg/mL for streptomycin, 8 µg/mL for chloramphenicol, 4 µg/mL for ampicillin, 32 µg/mL for tetracycline, and 1 µg/mL for erythromycin (45). We found that B^strR^ could adapt up to 256 µg/mL erythromycin, which is four times higher than that achieved by B^WT^. Similarly, B^strR^ could adapt up to 512 µg/mL tetracycline, which is eight times higher than that achieved by B^WT^. Notably, B^WT^ remains susceptible to doxycycline tolerating <1.6 µg/mL, even after 10 cycles of adaptive evolution, but the streptomycin-adapted variant B^strR^ could tolerate up to 64 µg/mL in fewer cycles of adaptive evolution, thereby gaining the ability to become resistant to doxycycline. Likewise, B^strR^ could tolerate five times higher dose of amoxicillin (64 µg/mL) than that achieved by B^WT^, while both B^WT^ and B^strR^ remained susceptible to piperacillin after laboratory adaptive evolution (Table 2; Fig. S1 and Table S2).

**TABLE 2:** Adaptation efficiency of wild-type (B^WT^) and streptomycin-adapted probiotics (B^strR^) to antibiotics^*a*^

Antibiotics cycle no.	Erythromycin cycle 11	Tetracycline cycle 7	Amoxicillin cycle 10	Doxycycline cycle 10	Piperacillin cycle 9
B^WT^	51.2–64.0 µg/mL Resistant	32.0–64.0 µg/mL Resistant	6.4–12 µg/mL Resistant	<1.6 µg/mL Susceptible	<1.6 µg/mL Susceptible
Antibiotics cycle no.	Erythromycin cycle 14	Tetracycline cycle 7	Amoxicillin cycle 5	Doxycycline cycle 7	Piperacillin cycle 8
B^strR^	128–256 µg/mL Resistant	256–512 µg/mL Resistant	51.2–64.0 µg/mL Resistant	32.0–64.0 µg/mL Resistant	<0.8 µg/mL Susceptible

^*a*^ Probiotics are assigned ‘resistance’ if they exceed the microbiological breakpoints for *Lactobacillus* strains listed in the EFSA guideline and ‘susceptible’ if they fall below the EFSA breakpoints.

### Transconjugant recipient bacteria detected after co-incubation with B^strR^

**Fig 3:**
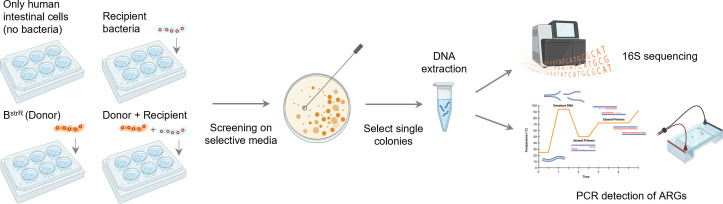
Workflow of the conjugative transfer experiments examined on human intestinal cells. Caco-2 or HCT-116 cells are incubated with probiotics adapted to 512 µg/mL streptomycin (B^strR^) and representative bacteria (recipient). Transconjugants are screened on streptomycin containing selective media. Colonies appearing on streptomycin containing media selective for the recipient bacteria represent transconjugants. Created with BioRender.com.

In our previous study, B^strR^ has been adapted from B^WT^ to tolerate up to 512 µg/mL streptomycin, and it also harbors ARGs responsible for streptomycin resistance, such as *strA*, *strB*, and *aadA* (Table 1). We, therefore, selected B^strR^ as the donor to investigate if streptomycin resistance can be transferred to representative bacteria *E. faecalis, Staphylococcus aureus*, and *E. coli* when co-incubated on human intestinal cells Caco-2 or HCT-116 (Fig. 3).

The donor and recipient bacteria were first examined on various growth media. We confirmed that the probiotics donor (B^strR^) could only grow on MRS media with and without 100 µg/mL streptomycin, but not on other media examined in this study (Fig. S2). We also confirmed that the mannitol salt agar (MSA) could only cultivate *E. faecalis*, while the Luria–Bertani (LB) media could cultivate both *E. coli* and *S. aureus*. All three recipient bacteria could not grow on media containing 100 µg/mL streptomycin. After co-incubation of recipient bacteria with the donor B^strR^ on Caco-2 or HCT-116 cells, screening on media selective for the recipient bacteria with and without streptomycin was performed. The appearance of bacteria colonies or transconjugants on the streptomycin containing selective media would indicate a successful transmission of streptomycin resistance from the donor B^strR^. Bacteria colonies appearing on 100 µg/mL streptomycin agar after co-incubation with B^strR^ and recipient were selected and cultured for DNA extractions, which were then subjected to PCR detection of ARGs (Fig. 3). As for the co-incubation of B^strR^ and *E. faecalis*, the DNA extracted from bacteria colonies appearing on the 100 µg/mL streptomycin MSA agar was also subjected to 16S sequencing to confirm the transconjugant identity.

**Fig 4:**
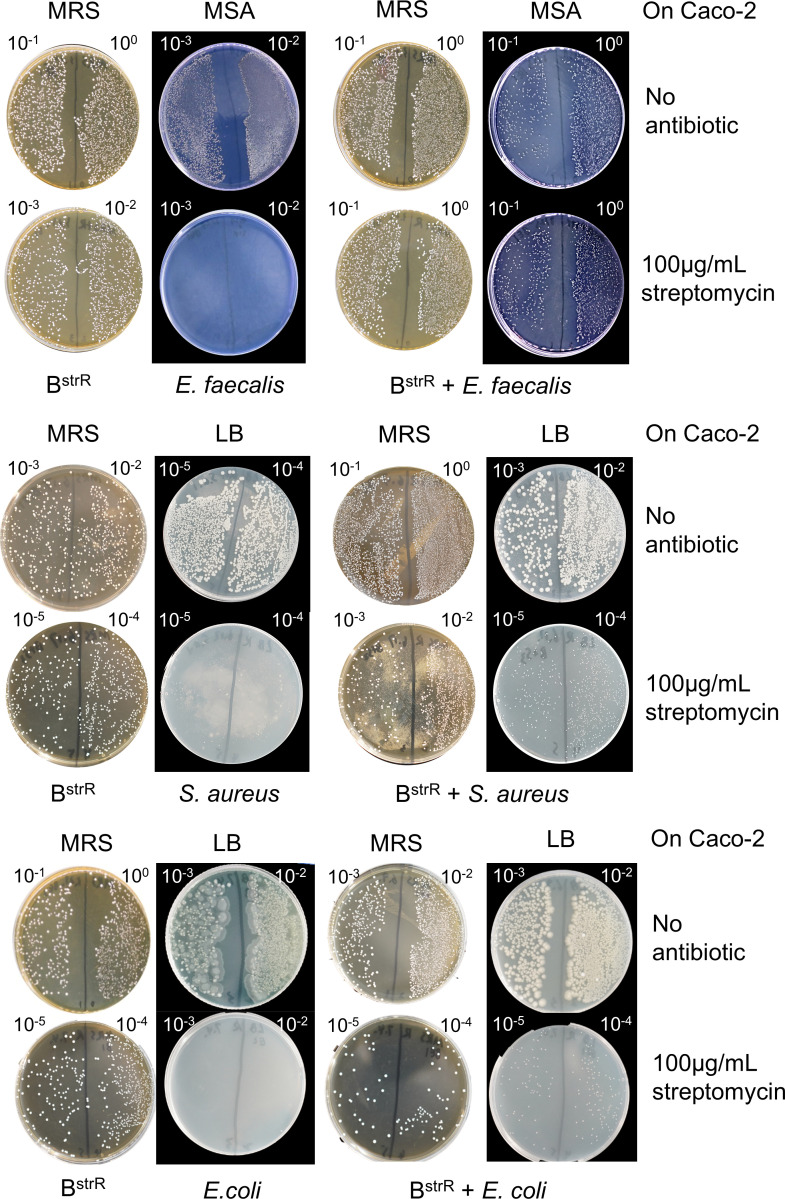
Screening for transconjugants on selective media after co-incubation of recipient bacteria with streptomycin-adapted probiotics B^strR^ on Caco-2 cells. Screening for transconjugants after co-incubation of B^strR^ and *E. faecalis* on HCT-116 cells is shown in Fig. S3. All recipient bacteria *E. faecalis*, *S. aureus*, or *E. coli* could not grow on streptomycin containing media (left panel). Transconjugants were detected on streptomycin containing media, which are selective for the recipient bacteria (right panel). B^strR^ can only grow on MRS media with and without streptomycin, but not on MSA or LB. The plates were divided into two halves, and the dilution factors that produce single bacteria colonies are indicated at the top of each plate. The agar plates showing different dilution factors correspond to about 50–200 CFUs used for bacteria enumeration and the estimation of transconjugant frequencies. Other dilution factors produced either too many or too few bacteria colonies, thus excluded from this figure. High-resolution images were captured by a digital camera, and the number of colonies was estimated using ImageJ (46).

Transconjugants were detected on streptomycin containing selective media after co-incubation of the recipient bacteria *E. faecalis, S. aureus*, or *E. coli* with B^strR^ on Caco-2 cells (Fig. 4). We also confirmed the presence of transconjugants when *E. faecalis was* co-incubated with B^strR^ on HCT-116 cells (Fig. S3). Moreover, the DNA extracted from three colonies appearing on the 100 µg/mL streptomycin MSA agar after the co-incubation of B^strR^ and *E. faecalis* on HCT-116 was confirmed through 16S sequencing to be that of the desired recipient bacteria. Similarly, two colonies from the 100 µg/mL streptomycin MSA agar after the same co-incubation treatment on Caco-2 was also confirmed through 16S sequencing to be *E. faecalis* (Table S3). To determine the ARG transfer frequency, high-resolution images were captured with a digital camera, and ImageJ (46) was used to estimate the number of colonies. The frequency of transconjugants was calculated by dividing the number of transconjugants with the number of recipients (22, 23).

Our data showed that the *E. faecalis* transconjugant frequency per recipient was 2.309 × 10^−6^ after co-incubation with B^strR^ on Caco-2 cells, while the transconjugant frequencies of *S. aureus* and *E. coli* were higher, recording an average of 3.615 × 10^−3^ and 6.014 × 10^−3^, respectively (Table 3). The same co-incubation experiment repeated on another cell line HCT-116 also yielded a similar frequency of the *E. faecalis* transconjugant (3.750 × 10^−6^) (Table 3).

**TABLE 3:** Transfer frequency of streptomycin resistance from B^strR^ to representative bacteria examined on human intestinal cell lines

Donor	Recipient	Cell line	Frequency of transconjugants	Frequency of transconjugants
			Individual experiments	Average ± SEM
B^strR^	*Enterococcus faecalis*	Caco-2	1.894 × 10^−6^, 2.143 × 10^−6^, 2.890 × 10^−6^	2.309 × 10^−6^ ± 2.995 × 10^−7^
B^strR^	*Enterococcus faecalis*	HCT-116	3.75 × 10^−6^, 1.463 × 10^−5^	9.188 × 10^−6^ ± 4.386 × 10^−6^
B^strR^	*Staphylococcus aureus*	Caco-2	6.168 × 10^−3^, 2.776 × 10^−3^, 1.901 × 10^−3^	3.615 × 10^−3^ ± 1.301 × 10^−3^
B^strR^	*Escherichia coli*	Caco-2	1.061 × 10^−2^, 1.377 × 10^−3^, 6.054 × 10^−3^	6.014 × 10^−3^ ± 2.665 × 10^−3^

### Transmission of ARGs from B^strR^ to recipient bacteria co-incubated on human cell lines

To investigate if the ARGs conferring resistance to streptomycin have been transferred from B^strR^ to the transconjugants, PCR was performed to detect the ARGs that were present in B^strR^ (Table 1). We did not detect any PCR products during the analysis using the DNA extracted from *E. faecalis*, *S. aureus*, and *E. coli*, thus confirming that these ARGs were absent in the recipient bacteria. After co-incubation with B^strR^ on Caco-2 cells, *aadA* was detected in the DNA of *E. coli* transconjugant, while *erm(B)−1* was detected in the DNA of *S. aureus* transconjugant. Both ARGs were detected in *E. faecalis* transconjugant after co-incubation with B^strR^ on HCT-116 cells but were undetected in *E. faecalis* transconjugant after co-incubation with B^strR^ on Caco-2 cells. Both *strA* and *strB* were undetected in all transconjugants (Table 4).

**TABLE 4:** ARGs detected in transconjugants after co-incubation with B^strR^

	Only recipient bacteria			Recipient bacteria co-incubated with B^strR^			
Cell lines	None			Caco-2			HCT-116
ARGs	*Enterococcus faecalis*	*Escherichia coli*	*Staphylococcus aureus*	*Enterococcus faecalis*	*Escherichia coli*	*Staphylococcus aureus*	*Enterococcus faecalis*
*aac(6′-aph(2*″)	×^*b*^	×	×	×	×	×	×
*^a^strA*	×	×	×	×	×	×	×
*^a^strB*	×	×	×	×	×	×	×
*tet(O*)	×	×	×	×	×	×	×
*tet(K*)	×	×	×	×	×	×	×
*tet(W*)	×	×	×	×	×	×	×
*^a^aadA*	×	×	×	×	√^*c*^	×	√
*erm(B)−1*	×	×	×	×	×	√	√
*bla*	×	×	×	×	×	×	×
*vanE*	×	×	×	×	×	×	×
*msrA/B*	×	×	×	×	×	×	×
*msrA*	×	×	×	×	×	×	×

^*a*^ Asterisks (*) represent ARGs known to confer resistance to streptomycin.

^*b*^ x means ARG undetected.

^*c*^ √ means ARG detected.

## DISCUSSION

Building on our previous study, which detected antibiotic resistant phenotypes of *Lactobacillus* probiotics from health supplements, we have, in this study, showed that they harbor ARGs known to confer resistance to multiple antibiotics, including tetracyclines, macrolides, aminoglycosides, and glycopeptides. Our data are consistent with many studies that detected antibiotic resistance in various probiotics, lactic acid bacteria, and/or *Lactobacillus* strains from humans, animals, fermented foods, and dairy products (9–11, 47, 48). Tetracycline resistant genes, especially the plasmid-borne *tet(K*) and *tet(M*), have been detected in *L. fermentum* strains from human feces and dairy products (49). Accordingly, *tetM*, *tetW*, *tetO*, *tetK*, and/or *tetL* were also detected in *Lactobacillus* from fermented foods (50, 51). Analysis of sequencing data sets and genome-wide annotations revealed that *tet(M*), together with *tet(W/N/W*), are the most common tetracycline-resistant genes found in lactic acid bacteria (23, 52, 53). However, we did not detect any tetracycline resistant genes in the recipient bacteria after co-incubation with probiotics harboring tetracycline-resistant genes (B^strR^). Resistance to erythromycin is one of the most common forms of macrolide resistance, and probiotic strains, such as *Lactobacillus rhamnosus* Pen and *L. rhamnosus* Oxy, have been shown to harbor *erm(A*), *erm(B*), and/or *erm(C*), which is not uncommon as these ARGs were also found in *L. fermentum* strains isolated from human feces and dairy products (49, 54). Acquired resistance to erythromycin can be conferred by *erm(B*), which has been reported in *Lactobacillus* from many sources (51, 55). Our study also confirmed its presence in *E. faecalis* and *S. aureus* transconjugants after co-incubation with probiotics (B^strR^). Other erythromycin resistant genes like *msrA/B* and *msrA* were also present in B^strR^, as well as other products, but they were not detected in the transconjugants. Many *Lactobacillus* not just from gut probiotic supplements but also from oral probiotic lozenges (13–16) have been shown to be phenotypically resistant to aminoglycosides, especially streptomycin and their ARGs, such as *ant(2″)-I*, *aph(3″)-III*, and *aadE*, have been reported (23). Consistently, we also detected many aminoglycoside ARGs in the probiotics tested in this study, including *aac(6′)-aph(2*″), *aph(3″)-III*, *strA*, and *strB*, which were all present in probiotics of product B, and this could account for its ability to adapt to high dose of streptomycin (B^strR^) (Table 1). Importantly, *aadA* was detected in *E. coli* and *E. faecalis* transconjugants after co-incubation with probiotics (B^strR^). Other ARGs responsible for resistance to rifampicin (*rpoB*), penicillin (*bla*), streptogramin (*vatC* and *vatE*), and vancomycin (*vanE*) detected in the probiotics tested in this study were also found in probiotics from other health supplements (14). Our genotypic data, therefore, affirmed the prevailing concern that the long-term consumption of antibiotic-resistant probiotics could adversely affect human health, especially with regard to the transmission of antimicrobial resistance.

Since probiotics are commonly prescribed following antibiotic treatments (39, 40), probiotics harboring ARGs may tolerate antibiotics at low doses and eventually climb a gradient of antibiotic. Indeed, recent studies have investigated the adaptive evolution of probiotics and leveraged whole-genome sequencing and comparative genomics to reveal the molecular strategies that enabled them to gain resistance to antibiotics. For instance, a dairy product-derived *L. plantarum* P-8 could adapt to 16 µg/mL ampicillin over 12 months of adaptative evolution by leveraging mechanisms involved in protein homeostasis, as the adapted bacteria showed altered carbon and amino acid metabolic pathways (36). Another study revealed that *L. casei* Zhang could tolerate up to 8 µg/mL amoxicillin and 32 µg/mL gentamycin after 10 months of adaptive evolution with a four-fold increase in the genome mutation frequency under either amoxicillin or gentamicin selection pressure compared with the no-antibiotic control groups (37). Likewise, *L. plantarum* ATCC14917 could tolerate up to 2,048 µg/mL streptomycin through laboratory adaptive evolution with mutation to a gene encoding for the S12 ribosomal protein being identified as crucial for a stable streptomycin-resistant phenotype (34).

**Fig 5:**
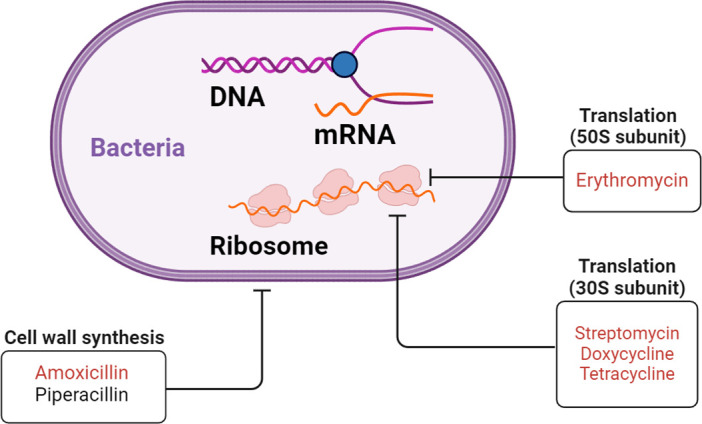
An illustration of the mechanism of action of antibiotics used in the laboratory adaptive evolution study. Antibiotics in red are those which the probiotics have gained resistance to through adaptive evolution. Created with BioRender.com.

Consistent with these studies, we have also showed in our previous study that probiotics from product B could climb a gradient of streptomycin to tolerate up to 512 µg/mL streptomycin (B^strR^) (15). Importantly, we hypothesized that the mutations accumulated in B^strR^ could afford probiotics better tolerance to other antibiotics, thus enabling them to more efficiently climb a gradient of antibiotics with similar classes or mechanism of actions. We, therefore, examined the adaptive evolution efficiency of B^strR^ to doxycycline, erythromycin, tetracycline, amoxicillin, and piperacillin and compared that to non-adapted probiotics, B^WT^. Except for piperacillin, B^strR^ tolerated high doses of all antibiotics compared to B^WT^ after adaptive evolution. Since tetracycline and doxycycline, much like streptomycin, target the 30S ribosomal subunit preventing aminoacyl-tRNA from binding to the bacterial ribosome (35), it is thus not surprising that B^strR^ adapted much more efficiently to these antibiotics due to plausible cross-protection afforded by the mutations already accumulated in B^strR^ (38, 56). Interestingly, tolerance to erythromycin and amoxicillin that target the 50S ribosomal subunit and cell wall synthesis, respectively, was also improved in B^strR^, which already harbored ARGs responsible for erythromycin and penicillin resistance (Fig. 5). This could be explained by the collective mutations already accumulated in the heterogeneous population of *Lactobacillus* in B^strR^ to offer mutualistic benefits, thus enabling them to tolerate antibiotics that are more distantly related (56). Moreover, the effect of other undetected ARGs or unknown genes capable of conferring tolerance to antibiotics in B^strR^ cannot be discounted. Future work that focuses on comparative whole-genome sequencing analysis detecting the single-nucleotide polymorphisms and structural variants in B^strR^ could, therefore, be revealing.

To examine the possibility of ARG transmission from probiotics to other bacteria, we co-incubated B^strR^ with two representative Gram-positive bacteria *E. faecalis* and *S. aureus* and one representative Gram-negative bacteria *E. coli. E. faecalis* and *E. coli* are integral members of the gut microflora and do not normally cause infections in healthy individuals, although certain strains may be pathogenic, especially to immunocompromised individuals (12, 57, 58). *S. aureus* predominantly resides on the skin and nasopharynx but can translocate to and colonizes the gut, resulting in enteritis or diarrhea (59). When co-incubated with B^strR^ on human intestinal cells, streptomycin resistance and, notably, the streptomycin-resistant gene, *aadA*, were detected in the *E. coli* and *E. faecalis* transconjugants. *aadA* encodes for an aminoglycoside nucleotidyltransferase that can inactivate streptomycin and is found on plasmids, transposons, or integrons (60). The erythromycin-resistant gene, *erm(B*), which was also detected in *E. faecalis* and *S. aureus* transconjugants, encodes a ribosomal methylase that causes target alternation of macrolide antibiotics and is found on plasmids in *Lactobacillus* (61, 62). Our co-incubation studies were conducted on human cells Caco-2 or HCT-116, which more closely resemble the surface properties of the human gut than that provided by filter mating. The *E. faecalis* transconjugant frequency per recipient was 2.31 × 10^−6^ and 9.188 × 10^−6^ on Caco-2 and HCT-116 cells, respectively, which was within the same range obtained by other co-incubation studies conducted using the filter mating approach. For instance, when *L. plantarum* and *L. rhamnosus* were used as donors of tetracycline resistance, the *E. faecalis* JH2-2 transconjugant frequency per recipient was 3.49 × 10^−6^ and 2.04 × 10^−6^, respectively (23). When conducted *in situ* on the food matrix, the *E. faecalis* JH2-2 transconjugant frequency per recipient was lower at 1.36 × 10^−8^ and 3.22 × 10^−7^, respectively (23). Another filter mating study reported a slightly higher *E. faecalis* transconjugant frequency of 1.39 × 10^−5^ when *L. plantarum* from fermented foods was used as a donor of tetracycline resistance (51). The same study also showed that when *L. fermentum* and *L. salivarius* were used as donors of erythromycin resistance, the *E. faecalis* transconjugant frequency was 2.62 × 10^−5^ and 2.9 × 10^−6^, respectively (51). In our co-incubation studies on Caco-2 cells, *S. aureus* and *E. coli* transconjugants were also detected but at higher frequencies per recipient of 3.615 × 10^−3^ and 6.014 × 10^−3^, respectively. Consistently, other *in vitro* co-culture studies also reported the transmission of the plasmid-borne quinolone-resistant gene, *qnrS*, and the tetracycline-resistant gene, *tetA*, from *Lactobacillus* to *E. coli* (25).

In-line with the broader goal of combating AMR, our data fill some existing research gaps and serve as a basis for further studies that contribute toward food safety, as well as in aiding the development of safer probiotics (63, 64). The detection of insertions and deletions and single-nucleotide polymorphisms in individual strains of wild-type and adapted probiotics would elucidate their molecular strategies for adaptation to high antibiotic concentrations. While multi-strain probiotic mixtures appear to confer greater health or clinical benefits than single-strain probiotics (65–67), the AMR impact and risk between these two are unclear. It is conceivable that multi-strain probiotic mixtures might offer synergistic effects that could favor bacteria adaptation to antibiotics and concomitantly also facilitate ARG transmission (68, 69). Follow-up comparative genomics and metagenomics studies conducted on animal models and in healthy, immunocompromised, and/or antibiotic-treated human cohorts will elucidate the molecular impact of probiotics on the gut resistome (19, 20), thus contributing to a more comprehensive understanding of probiotic consumption, application, and safety (64–72).

## MATERIALS AND METHODS

### Bacteria culture conditions and antibiotics used in this study

Probiotic supplements designated as products A, B, C, D, E, and F were purchased during our previous study (15). Based on the information on the product labels, all products, except for product F, contain heterogeneous probiotic strains mostly belonging to the *Lactobacillus* and *Bifidobacteria* genus. Product F contains only *L. reuteri* and is the only product in liquid form. The other products, whether capsule, tablet, or powder, were first ground. Next, 1 g of powdered samples was dissolved in 10 mL of phosphate buffered saline (PBS). A total of 10 µL dissolved samples were cultured overnight in 5 mL MRS broth at 37°C with shaking at 250 rpm for the enrichment of the *Lactobacillus* probiotic bacteria. Glycerol stocks of the mixed *Lactobacillus* culture (50%, v/v) were prepared for each sample and stored at −80°C (15). Bacteria were cultured under aerobic condition to preferentially cultivate *Lactobacillus* probiotic strains, which are facultative anaerobes as others, such as the obligate anaerobe *Bifidobacteria* that cannot grow as they would require a strict anaerobic condition and CO_2_ supplementation (41–43). The glycerol stock of the mixed *Lactobacillus* culture from product B (B^WT^) was used for the subsequent adaptive evolution studies to generate the streptomycin-adapted B^strR^.

The representative bacteria used in this study were *Escherichia coli* ATCC 25922, *Staphylococcus aureus* ATCC 25923, and *Enterococcus faecalis* ATCC 19433 obtained from BeNa Culture Collection (BNCC) (Beijing, China). They were all cultured under aerobic conditions at 37°C. *E. faecalis* used as recipient for co-incubation studies was cultured in the brain, heart, and infusion (BHI) growth medium, while *E. coli* and *S. aureus* recipients were cultured in Luria–Bertani (LB) broth. For the detection of transconjugants after co-incubation experiments, screening for the *E. coli* and *S. aureus* transconjugants was conducted on LB agar containing 100 µg/mL streptomycin, while the *E. faecalis* transconjugant was detected on mannitol salt agar (MSA) containing 100 µg/mL streptomycin.

Details of the antibiotics used in this study are summarized in Table 5. The antibiotics were dissolved in sterile Milli-Q water or ethanol to 10 mg/mL stock concentrations filtered through a filter unit of 0.2 µm pore size and stored at −20°C. Prior to the broth microdilution experiments, antibiotic stocks were diluted to 1 mg/mL and 0.1 mg/mL working concentrations, as required.

**TABLE 5:** Antibiotics used in this study

Antibiotic	Storage conditions	CAS no.	Lot no.	Brand (country)
Doxycycline hyclate	2–8°C	24390-14-5	C10075268	Macklin Biological Technology Ltd. (Shanghai, China)
Streptomycin sulfate	2–8°C	57-92-1	H11J10Z90149	Macklin Biological Technology Ltd. (Shanghai, China)
Erythromycin	RT	114–07-8	C14830299	Macklin Biological Technology Ltd. (Shanghai, China)
Amoxicillin trihydrate	2–8°C	61336-70-7	C12863062	Macklin Biological Technology Ltd. (Shanghai, China)
Piperacillin sodium	−20°C	59703-84-3	H2119223	Aladdin Scientific (Shanghai, China)
Tetracycline hydrochloride	<0°C	64-75-5	C13772430	Macklin Biological Technology Ltd. (Shanghai, China)

### PCR detection of ARGs

Bacteria DNA was isolated using GenElute Bacterial Genomic DNA Extraction Kit according to the manufacturer’s instructions (Sigma-Aldrich, Saint Louis, MO, USA). The quality of extracted DNA, such as its concentration and purity, was determined on a NanoDrop spectrophotometer (ND-ONE-W, ThermoFischer, WI, USA). A total of 48 primers specific for known ARGs and conferring resistance to 16 different antibiotics were purchased from Sangon Biotech (Shanghai, China) and used for the detection of ARGs from the bacterial DNA (Table S1). The PCR amplification program is an initial denaturation at 94°C for 5 min, followed by 30 cycles of 94°C for 45 s, annealing temperature for 45 s, and 72°C for 45 s, and a final extension of 10 min at 72°C. The PCR products were analyzed on 1% (w/v) agarose gel.

### 16S rRNA sequencing

The concentration and purity of extracted bacterial genomic DNA were determined on a NanoDropOne spectrophotometer (ND-ONE-W, ThermoFischer, WI) before being sent to Sangon Biotech (Shanghai, China) for PCR detection and sequencing of the 16S rRNA using the 27 F (5′-AGAGTTTGATCCTGGCTCAG-3′) and 1,492 R (5′-GGTTACCTTGTTACGACTT-3′) universal primers. Sequences of approximately 1,500 bp were compared to the NCBI GenBank database using blastn tool (73).

### Progressive adaptive evolution of probiotics to antibiotics

Progressive adaptive evolution studies were conducted according to previously described methods (15, 44). Briefly, probiotics selected based on prior antibiotic susceptibility test results were cultured in quadruplicates on 96-well plates containing a gradient of antibiotics. Wells with bacteria growth at least 50% of the no-antibiotic controls were selected for subcultures at that same antibiotic concentration for up to two generations before subjecting to the next gradient of antibiotic treatments. Probiotics were previously adapted up to 512 µg/mL streptomycin (B^strR^) after four cycles of adaptive evolution (15), and the adaptation efficiency of B^strR^ to other antibiotics was compared to that of the wild-type B^WT^ in terms of the number of cycles required to achieve tolerance to the same antibiotic concentration and/or the highest antibiotic concentration that could be tolerated by the probiotics.

### Conjugative transfer experiments on human intestinal cell lines

Human intestinal cell lines, Caco-2 and HCT-116, were selected for the co-incubation studies. The general maintenance of cell lines, cell culture, and conjugative transfer experiments were performed based on established procedures (74–78). Briefly, cells were grown in Dulbecco’s Modified Eagle Medium supplemented with 10% (v/v) fetal bovine serum (Gibco, ThermoFischer, WI, USA) and maintained at 37°C in a 5% CO_2_ incubator. The medium was changed in alternate days, and cells were passaged when they reached 80–90% confluency. Cells used for conjugative transfer experiments were at least 14 days old or have undergone seven to eight passages measured from the time of the first passage. Approximately 1 × 10^5^ mammalian cells were added to each well of a six-well plate containing growth medium and incubated at 37°C in a 5% CO_2_ cell culture incubator for 1 h prior to the addition of bacteria for conjugative transfer experiments. The streptomycin-adapted donor (B^strR^) and recipient bacteria (*E. faecalis*, *S. aureus*, or *E. coli*) were cultured for 1 day in their respective media: MRS for probiotics, BHI for *E. faecalis*, and LB for *E. coli* and *S. aureus*. The set-up for the conjugative transfer experiments was as follows: one group containing only the intestinal cells, one group containing intestinal cells plus 10^8^ donor bacteria B^strR^, one group containing intestinal cells plus 10^8^ recipient bacteria, and one group containing intestinal cells plus 10^8^ of both B^strR^ and recipient bacteria at a 1:1 ratio (Fig. 3). Every conjugative experiment was conducted in triplicate. The six-well plates were incubated for another 2 h at 37°C. The liquid media were carefully aspirated without disturbing the cells at the bottom of the wells, and 1 mL sterile PBS was used to gently wash down the bacteria from each well. The PBS mixture containing bacteria and cells was serially diluted to 10^0^, 10^1^, 10^2^, 10^3^, 10^4^, and 10^5^ and spread onto agar plates containing media selective for the recipient bacteria (MSA for *E. faecalis* and LB for *E. coli* and *S. aureus*) with and without 100 µg/mL streptomycin. Colonies that appear on the streptomycin containing agar plates with media selective for the recipient bacteria represent transconjugants. The agar plates were incubated for 48 h at 37°C, and high-resolution photographs of plates were captured with a digital camera for transconjugant frequency analysis on ImageJ (46).

## Data Availability

The original contributions presented in the study are included in the article or Supplemental Information; further inquiries can be directed to the corresponding authors.

## SUPPLEMENTAL MATERIAL

Supplemental material spectrum.00019-24-s0001.pdf Fig. S1 to S3; Tables S1 to S3.

ASM does not own the copyrights to Supplemental Material that may be linked to, or accessed through, an article. The authors have granted ASM a non-exclusive, world-wide license to publish the Supplemental Material files. Please contact the corresponding author directly for reuse.
