# Supplementary material for: Evaluating the health risk of probiotic supplements from the perspective of antimicrobial resistance
Source: Microbiol Spectr. 2024 Dec 10;13(1):e00019-24. doi: 10.1128/spectrum.00019-24 (PMC11705942; doi:10.1128/spectrum.00019-24)
Supplement: Supplemental material — Fig. S1 to S3; Tables S1 to S3. [file spectrum.00019-24-s0001.pdf]

SUPPLEMENTAL MATERIAL

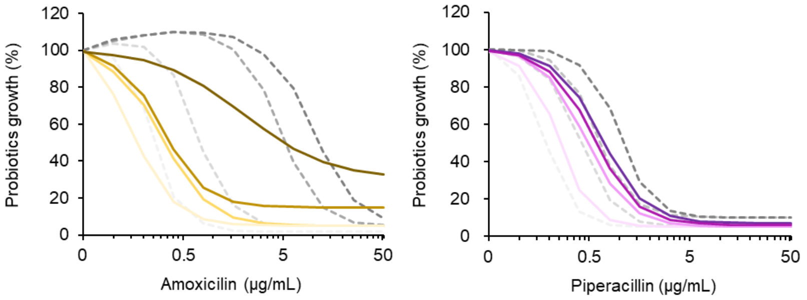

FIG S1 Adaptive evolution of probiotics to antibiotics. The non-adapted and streptomycin-adapted probiotics B^{WT} and B^{strR}, are represented as dotted and solid lines, respectively. There is no clear difference in adaptation efficiency of B^{strR} to piperacillin compared to the non-adapted probiotics B^{WT}. Line color intensities represent increasing number of adaptive evolution cycle. Dose response curves were generated from data points of four biological replicates for each antibiotic concentration. Raw data of each cycle represented in this figure, the average, standard deviation (SD) and standard error of the mean (SEM), are shown in Table S2 and colored corresponding to the line colors in this figure.

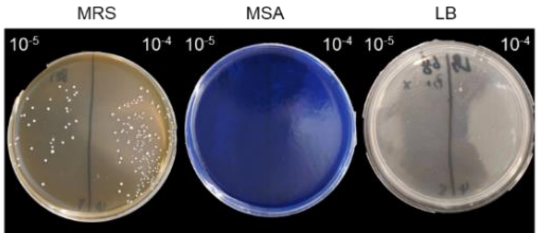

FIG S2 *Lactobacillus* containing B^{strR} grow on MRS agar displaying opaque white colonies, but not on MSA and LB agar. Five microliters B^{strR} glycerol stock was cultured overnight at 37°C in 1mL MRS broth with shaking at 250rpm. Fifty microliters of 10^{-4} (right panel) and 10^{-5} (left panel) diluted B^{strR} overnight culture was spread onto MRS, MSA and LB agar. The agar plates were incubated at 37°C for 48h.

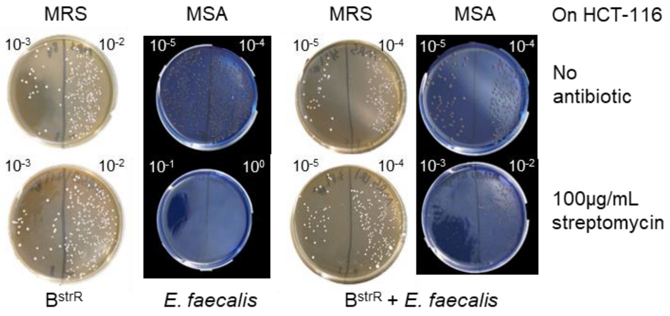

FIG S3 Screening for transconjugants on selective media after co-incubation of recipient bacteria *E. faecalis* with streptomycin-adapted probiotics B^{strR} on HCT-116 cells. *E. faecalis* could not grow on streptomycin containing media (left panel). Transconjugants were detected on streptomycin containing media which are selective for the recipient bacteria (right panel). B^{strR} can only grow on MRS media with and without streptomycin, but not on MSA or LB. The plates were divided into two halves and the dilution factors producing single colonies are indicated at the top

of each plate. The agar plates showing different dilution factors correspond to about 50-200 CFUs which was used for bacteria enumeration and the estimation of transconjugant frequencies. Other dilution factors produced either too many or too few bacteria colonies, thus excluded from this figure. High-resolution images were captured by a digital camera and the number of colonies were estimated using ImageJ (43).

Table S1 Primers for PCR detection of known ARGs.

Antibiotic	ARG	Primers 5'-3' (Forward; Reverse)	Product size (bp)
Gentamicin	*aac(6')-aph(2'')*	CCAAGAGCAATAAGGGCATA; CACTATCATAACCACTACCG	240
	aac(6')Ieaph(2'')Ia	CAGAGCCTTGGGAAGATGAAG; CCTCGTGTAATTCATGTTCTGGC	348
Streptomycin	*strA*	CTTGGTGATAACGGCAATTC; CCAATCGCAGATAGAAGGC	548
	strB	ATCGTCAAGGGATTGAAACC; GGATCGTAGAACATATTGGC	509
	*aadA**	ATCCTTCGGCGCGATTTTG; GCAGCGCAATGACATTCTTG	744*
	aadE	ATGGAATTATTCCCACCTGA; TCAAAACCCCTATTAAAGCC	565
	ant(6)	ACTGGCTTAATCAATTTGGG; GCCTTTCCGCCACCTCACCG	597
Kanamycin	*aph(3'')-III*	GCCGATGTGGATTGCGAAAA; GCTTGATCCCCAGTAAGTCA	292
	ant(2'')-I	GGGCGCGTCATGGAGGAGTT; TATCGCGACCTGAAAGCGGC	329
Neomycin	*aph(3'')-I*	AACGTCTTGCTCGAGGCCGCG; GGCAAGATCCTGGTATCGGTCTGCG	670
	aph(3'')-III	GCCGATGTGGATTGCGAAAA; GCTTGATCCCCAGTAAGTCA	292
Tetracycline	*tet(M)*	GGTGAACATCATAGACACGC; CTTGTTCGAGTTCCAATGC	401
	tet(K)	TCGATAGGAACAGCAGTA; CAGCAGATCCTACTCCTT	169
	tet(W)	GAGAGCCTGCTATATGCCAGC; GGGCGTATCCACAATGTTAAC	168
	tet(L)	GTMGTTGCGCGCTATATTCC; GTGAAMGRWAGCCCACCTAA	696
	tet(O)	AATGAAGATTCCGACAATTT; CTCATGCGTTGTAGTATTCCA	781
	tet(S)	ATCAAGATATTAAGGAC; TTCTCTATGTGGTAATC	573
Erythromycin	*erm(A)*	AAGCGGTAAACCCCTCTGAG; TCAAAGCCTGTCGGAATTGG	441
	*erm(B)-1***	CATTTAACGACGAAACTGGC; GGAACATCTGTGGTATGGCG	639**
	erm(C)	TCAAAACATAATATAGATAAA; GCTAATATTGTTTAAATCGTCAAT	642
	erm(T)	TATTATTGAGATTGGTTCAGGG; GGATGAAAGTATTCTCTAGGGATTT	395
	mefA	AGTATCATTAATCACTAGTGC; TTCTTCTGGTACTAAAAGTGG	348
Clindamycin	*lnu(A)*	GGTGGCTGGGGGGTAGATGTATTAACTGG; GCTTCTTTTGAAATACATGGTATTTTTCGATC	323
	lnu(B)	CCTACCTATTGTTTGTGGAA; ATAACGTTACTCTCCTATTTC	925
Chloramphenicol	*catA*	GGATATGAAATTTATCCCTC; CAATCATCTACCCTATGAAT	486
	cat	TTAGGTTATTGGGATAAGTTA; GCATGRTAACCATCACAWAC	300
	cat-TC	CAATAGCGACGGAGAGTTAGG; AATCCTGCATGATAACCATCAC	384
Ampicillin	*blaZ*	ACTTCAACACCTGCTGCTTTC; TAGGTTCAGATTGGCCCTTAG	240
	bla	CATARTTCCGATAATASMGCC; CGTSTTTAACTAAGTATSGY	297
	mecA	GGGATCATAGCGTCATTATTC; AGTTCTGCAGTACCGGATTTGC	1429
Vancomycin	*vanA*	ATGAATAGAATAAAAGTTGCAATAC; CCCCTTTAACGCTAATACGAT	1028
	vanB	CCCGAATTTCAAATGATTGAAAA; CGCCATCCTCCTGCAAAA	457
	vanC	GCTGAAATATGAAGTAATGACCA; CGGCATGGTGTTGATTTCGTT	832
	vanE	TGTGGTATCGGAGCTGCAG; GTCGATTCTCGCTAATCC	513
	vanX	TCGCGGTAGTCCCACCATTCGTT; AAATCATCGTTGACCTGCGTTAT	454
Quinupristin/	*vatC*	GAAATGGTTGGGAGAAGCATACC; CAGCAATCGCGCCCGTTTG	392
Dalfopristin	*vatE*	CTATACCTGACGCAAATGC; GGTTCAAATCTTGGTCCG	490
Linezolid	*cfr*	TGAAGTATAAAGCAGGTTGGGAGTCA; ACCATATAATTGACCACAAGCAGC	746
Trimethoprim	*dfrA*	CTTTTCTACGCACTAAATGTAAG; CATTATCAATAATTGTCGCTCAC	474
	dfrD	GGAAGGGCTTTACCTGACAGAAG; CGACATAAGGCAAGAACATAACATA	175
Rifampicin	*rpoB*	TAACCGTGGTGCTTGGCTDGAATWYGAAAC; ATCAAACCAATGTTAGGNCCTTCWGGDGTTTC	1100
Ciprofloxacin	*gyrA*	GAYTATGCWATGTCAGTTATTGT; GGAATRTTRGAYGTCATACCAAC	286
	parC	TATTCYAAATAYATCATTCARGA; GCYTCNGTATAACGCATMGCCG	286
	int	GCGTGATTGTATCTCACT; GACGCTCCTGTTGCTTCT	1028
Macrolide	*msrA/B*	GCAAATGGTGTAGGTAAGACAACT; ATCATGTGATGTAAACAAAAT	399
antibiotics	*msrA*	GGCACAATAAGAGTGTTTAAAGG; AAGTTATATCATGAATAGATTGTCCTGTT	939
	msrC	AAGGAATCCTTCTCTCTCCG; GTAAACAAAATCGTTCCCG	343
	Tn554	AAGCGGTAAACCCCTCTGAG; TCAAAGCCTGTCGGAATTGG	440

The table is adapted from Wang et al. (2022), J Oral Microbiol 14(1):2019992 which contains all the original sources of the ARGs except for *aadA* (*) which is obtained from Yuan et al. (2019), Front Microbiol 10:1749, and *erm(B)-1* (**) which is obtained from Brenciani et al. (2007), Antimicrob Agents Chemother 51(4), 1209-1216.

Table S2. Dose response curve data

ERYTHROMYCIN

*Only the raw data for the cycles represented in Figure 2 are shown in this table

WT cycle 1 (------)																																												
Dose (µg/mL)	0.000	0.000	0.000	0.000	0.100	0.100	0.100	0.100	0.200	0.200	0.200	0.200	0.400	0.400	0.400	0.400	0.800	0.800	0.800	0.800	1.600	1.600	1.600	1.600	3.200	3.200	3.200	3.200	6.400	6.400	6.400	6.400	12.800	12.800	12.800	12.800	25.600	25.600	25.600	25.600	51.200	51.200	51.2	51.2
% bacteria growth	99.952	105.020	96.488	98.540	99.695	101.363	98.605	99.246	101.877	101.235	95.204	101.748	102.197	102.967	98.861	101.171	96.167	103.545	93.023	90.136	74.290	84.298	40.225	32.719	15.910	7.442	8.083	12.189	10.521	5.774	10.136	14.435	14.370	2.694	13.601	19.503	11.740	1.347	11.997	14.755	6.351	2.502	11.291	12.638
AVE		100.000				99.727				100.016				101.299				95.718				57.883				10.906				10.217				12.542				9.960				8.196		
SD		3.636				1.179				3.220				1.784				5.770				25.246				3.944				3.542				7.068				5.902				4.660		
SEM		1.818				0.589				1.610				0.892				2.885				12.623				1.972				1.771				3.534				2.951				2.330		
WT cycle 3 (------)																																												
Dose (µg/mL)	0.000	0.000	0.000	0.000	0.100	0.100	0.100	0.100	0.200	0.200	0.200	0.200	0.400	0.400	0.400	0.400	0.800	0.800	0.800	0.800	1.600	1.600	1.600	1.600	3.200	3.200	3.200	3.200	6.400	6.400	6.400	6.400	12.800	12.800	12.800	12.800	25.600	25.600	25.600	25.600	51.200	51.200	51.200	51.200
% bacteria growth	96.261	107.057	95.597	101.086	100.905	104.765	106.333	104.282	103.559	104.885	102.955	104.222	104.765	104.885	101.448	108.745	104.584	102.895	102.714	102.171	90.531	106.634	100.181	109.590	85.464	80.579	33.112	110.012	15.440	11.882	13.390	9.952	20.265	10.736	15.380	16.405	35.826	11.098	15.380	22.256	10.856	24.367	45.597	-6.454
AVE		100.000				104.071				103.905				104.961				103.091				101.734				77.292				12.666				15.697				21.140				18.592		
SD		5.302				2.285				0.833				2.984				1.042				8.439				32.146				2.324				3.919				10.816				21.983		
SEM		2.651				1.143				0.417				1.492				0.521				4.220				16.073				1.162				1.960				5.408				10.991		
WT cycle 6 (------)																																												
Dose (µg/mL)	0.000	0.000	0.000	0.000	0.100	0.100	0.100	0.100	0.200	0.200	0.200	0.200	0.400	0.400	0.400	0.400	0.800	0.800	0.800	0.800	1.600	1.600	1.600	1.600	3.200	3.200	3.200	3.200	6.400	6.400	6.400	6.400	12.800	12.800	12.800	12.800	25.600	25.600	25.600	25.600	51.200	51.200	51.200	51.200
% bacteria growth	95.050	99.261	104.353	101.336	100.833	104.165	103.976	96.495	105.548	104.542	105.673	109.068	72.041	105.736	41.930	109.822	105.673	106.554	106.616	102.845	104.856	104.730	104.416	106.491	98.444	102.342	98.821	95.175	38.535	29.860	24.202	66.132	21.939	9.744	30.992	13.138	20.682	23.259	29.797	28.414	19.299	13.201	17.413	12.824
AVE		100.000				101.367				106.208				82.383				105.422				105.123				98.696				39.683				18.953				25.538				15.684		
SD		3.907				3.589				1.973				31.842				1.771				0.930				2.931				18.592				9.530				4.289				3.183		
SEM		1.953				1.795				0.986				15.921				0.886				0.465				1.465				9.296				4.765				2.144				1.592		
WT cycle 8 (------)																																												
Dose (µg/mL)	0.000	0.000	0.000	0.000	0.100	0.100	0.100	0.100	0.200	0.200	0.200	0.200	0.400	0.400	0.400	0.400	0.800	0.800	0.800	0.800	1.600	1.600	1.600	1.600	3.200	3.200	3.200	3.200	6.400	6.400	6.400	6.400	12.800	12.800	12.800	12.800	25.600	25.600	25.600	25.600	51.200	51.200	51.200	51.200
% bacteria growth	102.147	103.000	98.279	96.574	100.901	103.852	99.918	96.837	103.590	105.294	99.787	101.623	106.081	107.786	104.442	103.524	99.394	109.621	103.983	96.574	104.770	99.459	95.919	103.655	98.345	105.556	100.508	100.246	97.361	98.213	95.525	94.411	95.329	88.051	81.954	87.658	66.874	59.138	55.729	59.662	15.866	25.832	18.489	18.030
AVE		100.000				100.377				102.573				105.458				102.393				100.951				101.164				96.378				88.248				60.351				19.554		
SD		3.072				2.892				2.388				1.878				5.705				4.060				3.083				1.726				5.481				4.686				4.339		
SEM		1.536				1.446				1.194				0.939				2.852				2.030				1.542				0.863				2.741				2.343				2.169		
WT cycle 11 (------)																																												
Dose (µg/mL)	0.000	0.000	0.000	0.000	0.100	0.100	0.100	0.100	0.200	0.200	0.200	0.200	0.400	0.400	0.400	0.400	0.800	0.800	0.800	0.800	1.600	1.600	1.600	1.600	3.200	3.200	3.200	3.200	6.400	6.400	6.400	6.400	12.800	12.800	12.800	12.800	25.600	25.600	25.600	25.600	51.200	51.200	51.200	51.200
% bacteria growth	94.429	94.364	106.318	104.889	105.214	104.109	85.139	100.536	102.095	83.969	91.701	106.253	76.368	99.497	84.229	81.955	81.371	91.051	86.373	95.923	104.629	92.285	92.805	95.144	97.223	93.974	89.232	107.422	94.624	89.687	92.740	96.833	97.353	88.972	89.816	104.369	86.763	79.682	64.414	72.925	54.540	26.994	49.212	26.279
AVE		100.000				98.749				96.005				85.512				88.680				96.216				96.963				93.471				95.127				75.946				39.256		
SD		6.497				9.291				10.091				9.891				6.241				5.745				7.707				3.027				7.222				9.541				14.736		
SEM		3.248				4.645				5.046				4.945				3.120				2.872				3.853				1.513				3.611				4.770				7.368		
BstrR cycle 4 (——)																																												
Dose (µg/mL)	0.000	0.000	0.000	0.000	0.100	0.100	0.100	0.100	0.200	0.200	0.200	0.200	0.400	0.400	0.400	0.400	0.800	0.800	0.800	0.800	1.600	1.600	1.600	1.600	3.200	3.200	3.200	3.200	6.400	6.400	6.400	6.400	12.800	12.800	12.800	12.800	25.600	25.600	25.600	25.600	51.200	51.200	51.200	51.200
% bacteria growth	97.805	100.776	97.541	103.879	99.257	97.937	96.551	107.642	98.399	97.805	101.040	103.680	99.455	100.380	103.284	103.416	101.436	99.917	99.389	100.512	94.108	97.739	96.220	99.851	10.266	44.133	11.916	4.984	5.446	4.258	2.476	2.476	1.948	2.278	3.994	3.070	2.938	2.080	2.278	3.268	0.627	2.608	0.891	2.806
AVE		100.000				100.347				100.231				101.634				100.314				96.980				17.825				3.664				2.822				2.641				1.733		
SD		2.973				4.987				2.695				2.018				0.877				2.425				17.786				1.455				0.912				0.556				1.132		
SEM		1.486				2.494				1.348				1.009				0.439				1.213				8.893				0.728				0.456				0.278				0.566		
BstrR cycle 6 (——)																																												
Dose (µg/mL)	0.000	0.000	0.000	0.000	0.100	0.100	0.100	0.100	0.200	0.200	0.200	0.200	0.400	0.400	0.400	0.400	0.800	0.800	0.800	0.800	1.600	1.600	1.600	1.600	3.200	3.200	3.200	3.200	6.400	6.400	6.400	6.400	12.800	12.800	12.800	12.800	25.600	25.600	25.600	25.600	51.200	51.200	51.200	51.200
% bacteria growth	95.897	103.476	103.163	97.463	104.291	103.790	105.230	104.040	103.226	102.850	101.785	103.727	103.664	105.606	101.723	90.135	102.161	104.291	101.660	94.206	97.025	102.913	95.835	101.221	99.280	103.602	100.720	100.532	91.074	95.146	89.884	88.256	70.717	96.649	73.724	58.315	17.350	14.093	9.897	8.707	13.279	9.521	7.830	9.709
AVE		100.000				104.338				102.897				100.282				100.579				99.248				101.034				91.090				74.851				12.512				10.085		
SD		3.888				0.629				0.824				6.948				4.399				3.362				1.828				2.940				15.989				3.968				2.291		
SEM		1.944				0.315				0.412				3.474				2.200				1.681				0.914				1.470				7.995				1.984				1.146		
BstrR cycle 11 (——)																																												
Dose (µg/mL)	0.000	0.000	0.000	0.000	1.000	1.000	1.000	1.000	2.000	2.000	2.000	2.000	4.000	4.000	4.000	4.000	8.000	8.000	8.000	8.000	16.000	16.000	16.000	16.000	32.000	32.000	32.000	32.000	64.000	64.000	64.000	64.000	######	######	######	128.000	######	######	######	######	######	######	######	######
% bacteria growth	97.618	106.605	100.321	95.456	102.280	117.011	109.848	105.591	117.754	116.065	97.753	98.632	109.646	112.686	101.064	86.334	114.240	99.375	97.618	86.199	105.727	77.212	90.591	73.090	67.887	61.536	47.211	47.076	20.116	8.696	21.062	31.062	17.210	13.426	24.913	28.427	14.237	15.521	26.332	30.183	18.967	23.089	29.778	34.305
AVE		100.000				108.683				107.551				102.433				99.358				86.655				55.928				20.234				20.994				21.568				26.535		
SD		4.832				6.358				10.834				11.807				11.513				14.747				10.469				9.148				6.885				7.900				6.832		
SEM		2.416				3.179				5.417				5.903				5.757				7.373				5.235				4.574				3.442				3.950				3.416		
BstrR cycle 12 (——)																																												
Dose (µg/mL)	0.000	0.000	0.000	0.000	1.000	1.000	1.000	1.000	2.000	2.000	2.000	2.000	4.000	4.000	4.000	4.000	8.000	8.000	8.000	8.000	16.000	16.000	16.000	16.000	32.000	32.000	32.000	32.000	64.000	64.000	64.000	64.000	######	######	######	128.000	######	######	######	######	######	######	######	######
% bacteria growth	103.199	97.173	101.562	98.066	105.579	102.381	106.546	110.935	104.463	101.934	104.761	108.257	99.926	89.511	101.339	106.770	86.907	101.711	101.265	107.960	100.298	93.379	107.216	111.382	98.735	86.610	100.744	85.345	81.923	44.876	75.823	107.290	-4.519	-1.990	10.433	26.502	-7.049	-3.255	11.624	23.972	8.053	3.961	20.922	25.460
AVE		100.000				106.360				104.854				99.386				99.461				103.069				92.858				77.478				7.606				6.323				14.599		
SD		2.852				3.532				2.599				7.214				8.910				7.914				8.005				25.652				14.191				14.261				10.230		
SEM		1.426				1.766				1.300				3.607				4.455				3.957				4.002				12.826				7.095				7.131				5.115		
BstrR cycle 14 (——)																																												
Dose (µg/mL)	0.000	0.000	0.000	0.000	1.000	1.000	1.000	1.000	2.000	2.000	2.000	2.000	4.000	4.000	4.000	4.000	8.000	8.000	8.000	8.000	16.000	16.000	16.000	16.000	32.000	32.000	32.000	32.000	64.000	64.000	64.000	64.000	######	######	######	128.000	######	######	######	######	######	######	######	######
% bacteria growth	101.248	104.166	99.692	94.894	99.562	102.869	101.961	99.108	100.146	102.091	101.248	97.941	96.645	94.764	94.310	95.218	88.669	99.822	96.255	93.662	95.024	95.088	95.088	94.116	85.103	86.400	93.921	93.338	71.746	91.457	89.836	76.220	65.262	74.210	59.556	41.465	20.198	5.738	7.165	8.656	16.956	11.315	12.158	10.990
AVE		100.000				100.875				100.357				95.234				94.602				94.829				89.690				82.315				60.123				10.439				12.855		
SD		3.876				1.826				1.796				1.011				4.693				0.476				4.585				9.815				13.824				6.614				2.778		
SEM		1.938				0.913				0.898				0.505				2.346				0.238				2.293				4.908				6.912				3.307				1.389		

Table S2. Dose response curve data

TETRACYCLINE

*Only the raw data for the cycles represented in Figure 2 are shown in this table

WT cycle 1 (------)

Dose (µg/mL)	0.000	0.000	0.000	0.000	1.000	1.000	1.000	1.000	2.000	2.000	2.000	2.000	4.000	4.000	4.000	4.000	8.000	8.000	8.000	8.000	16.000	16.000	16.000	16.000	32.000	32.000	32.000	32.000	64.000	64.000	64.000	64.000	128.000	128.000	128.000	128.000	256.000	256.000	256.000	256.000	512.000	512.000	512.000	512.000
% bacteria growth	100.000	102.712	99.186	98.102	87.458	98.712	95.729	89.492	87.390	92.339	87.051	89.220	84.339	83.525	81.424	81.966	62.169	71.186	57.356	25.831	9.288	6.983	0.407	-0.271	5.966	-1.898	-1.220	0.271	3.186	-0.407	-1.831	0.339	4.746	-3.458	-4.746	-3.661	-0.068	-2.915	-1.220	1.627	2.508	0.678	8.000	4.475
AVE	100.000				92.847				89.000				82.814				54.136				4.102				0.780				0.322				-1.780				-0.644				3.915			
SD	1.968				5.260				2.421				1.352				19.722				4.760				3.574				2.111				4.387				1.913				3.134			
SEM	0.984				2.630				1.211				0.676				9.861				2.380				1.787				1.056				2.193				0.957				1.567			

WT cycle 3 (------)

Dose (µg/mL)	0.000	0.000	0.000	0.000	1.000	1.000	1.000	1.000	2.000	2.000	2.000	2.000	4.000	4.000	4.000	4.000	8.000	8.000	8.000	8.000	16.000	16.000	16.000	16.000	32.000	32.000	32.000	32.000	64.000	64.000	64.000	64.000	128.000	128.000	128.000	128.000	256.000	256.000	256.000	256.000	512.000	512.000	512.000	512.000
% bacteria growth	98.953	100.371	101.857	98.818	98.818	102.668	101.722	99.966	101.925	102.668	98.075	100.574	100.507	96.049	96.995	102.262	66.198	88.553	92.402	71.736	21.760	107.193	75.248	104.424	66.131	54.110	59.107	53.367	30.540	28.581	26.150	25.137	14.939	13.588	16.627	15.277	15.479	6.159	-2.013	12.237	-1.202	9.806	9.401	-12.008
AVE	100.000				100.794				100.810				98.953				79.722				77.156				58.179				27.602				15.108				7.966				1.499			
SD	1.424				1.728				2.019				2.923				12.720				39.657				5.883				2.434				1.248				7.693				10.347			
SEM	0.712				0.864				1.009				1.462				6.360				19.829				2.941				1.217				0.624				3.846				5.173			

WT cycle 4 (------)

Dose (µg/mL)	0.000	0.000	0.000	0.000	1.000	1.000	1.000	1.000	2.000	2.000	2.000	2.000	4.000	4.000	4.000	4.000	8.000	8.000	8.000	8.000	16.000	16.000	16.000	16.000	32.000	32.000	32.000	32.000	64.000	64.000	64.000	64.000	128.000	128.000	128.000	128.000	256.000	256.000	256.000	256.000	512.000	512.000	512.000	512.000
% bacteria growth	100.395	99.573	99.699	100.332	98.181	98.181	97.675	91.980	98.624	98.877	98.877	96.852	97.991	96.536	96.536	95.777	93.183	93.499	90.968	69.266	76.479	77.491	74.771	65.849	25.293	63.761	54.650	41.110	7.260	9.791	4.287	5.362	6.058	2.642	1.629	0.997	2.262	1.882	0.680	1.756	3.464	3.021	2.705	3.717
AVE	100.000				96.504				98.307				96.710				86.729				73.648				46.204				6.675				2.831				1.645				3.227			
SD	0.424				3.025				0.977				0.926				11.696				5.319				16.761				2.414				2.255				0.678				0.451			
SEM	0.212				1.513				0.489				0.463				5.848				2.659				8.381				1.207				1.128				0.339				0.226			

WT cycle 7 (------)

Dose (µg/mL)	0.000	0.000	0.000	0.000	1.000	1.000	1.000	1.000	2.000	2.000	2.000	2.000	4.000	4.000	4.000	4.000	8.000	8.000	8.000	8.000	16.000	16.000	16.000	16.000	32.000	32.000	32.000	32.000	64.000	64.000	64.000	64.000	128.000	128.000	128.000	128.000	256.000	256.000	256.000	256.000	512.000	512.000	512.000	512.000
% bacteria growth	95.165	98.497	99.952	106.375	88.984	95.528	99.043	97.467	96.316	98.012	96.801	103.466	86.196	93.044	95.892	104.920	89.590	89.408	90.135	83.348	81.106	77.774	87.833	88.560	50.870	62.019	74.744	74.441	47.416	67.109	27.056	52.809	-2.030	18.997	46.628	35.721	-1.666	9.786	19.481	30.631	-11.362	10.513	24.026	36.933
AVE	99.997				95.255				98.649				95.013				88.120				83.818				65.518				48.597				24.829				14.558				15.028			
SD	4.700				4.421				3.290				7.758				3.196				5.244				11.425				16.592				21.207				13.767				20.636			
SEM	2.350				2.211				1.645				3.879				1.598				2.622				5.712				8.296				10.604				6.883				10.318			

BstrR cycle 1 (——)

Dose (µg/mL)	0.000	0.000	0.000	0.000	1.000	1.000	1.000	1.000	2.000	2.000	2.000	2.000	4.000	4.000	4.000	4.000	8.000	8.000	8.000	8.000	16.000	16.000	16.000	16.000	32.000	32.000	32.000	32.000	64.000	64.000	64.000	64.000	128.000	128.000	128.000	128.000	256.000	256.000	256.000	256.000	512.000	512.000	512.000	512.000
% bacteria growth	105.628	104.249	98.540	91.583	100.246	99.918	96.308	92.961	99.393	97.490	90.205	91.911	91.124	95.849	87.777	89.943	70.320	68.942	68.285	77.473	88.171	56.669	72.158	50.172	39.147	41.181	21.034	29.434	14.733	12.633	11.386	8.302	15.783	-0.361	6.661	13.290	6.333	0.295	7.646	7.514	17.621	10.139	9.746	12.305
AVE	100.000				97.358				94.750				91.173				71.255				66.792				32.699				11.764				8.843				5.447				12.453			
SD	6.395				3.432				4.389				3.412				4.231				16.976				9.314				2.690				7.244				3.485				3.625			
SEM	3.198				1.716				2.194				1.706				2.116				8.488				4.657				1.345				3.622				1.742				1.812			

BstrR cycle 2 (——)

Dose (µg/mL)	0.000	0.000	0.000	0.000	1.000	1.000	1.000	1.000	2.000	2.000	2.000	2.000	4.000	4.000	4.000	4.000	8.000	8.000	8.000	8.000	16.000	16.000	16.000	16.000	32.000	32.000	32.000	32.000	64.000	64.000	64.000	64.000	128.000	128.000	128.000	128.000	256.000	256.000	256.000	256.000	512.000	512.000	512.000	512.000
% bacteria growth	100.856	99.934	97.563	101.646	93.612	92.361	94.007	99.539	91.044	85.249	88.936	99.210	82.878	81.956	81.692	90.122	25.453	22.226	17.089	22.028	9.384	-1.284	11.492	23.543	0.296	-0.428	10.372	23.609	0.099	-0.428	11.426	24.465	-0.033	0.428	13.467	25.058	-0.099	1.548	15.772	26.309	1.482	5.301	19.526	28.416
AVE	100.000				94.880				91.110				84.162				21.699				10.784				8.462				8.890				9.730				10.882				13.681			
SD	1.769				3.184				5.907				4.006				3.451				10.180				11.237				11.735				11.983				12.511				12.521			
SEM	0.884				1.592				2.954				2.003				1.725				5.090				5.618				5.867				5.991				6.256				6.261			

BstrR cycle 6 (——)

Dose (µg/mL)	0.000	0.000	0.000	0.000	1.000	1.000	1.000	1.000	2.000	2.000	2.000	2.000	4.000	4.000	4.000	4.000	8.000	8.000	8.000	8.000	16.000	16.000	16.000	16.000	32.000	32.000	32.000	32.000	64.000	64.000	64.000	64.000	128.000	128.000	128.000	128.000	256.000	256.000	256.000	256.000	512.000	512.000	512.000	512.000
% bacteria growth	103.358	98.613	100.351	97.678	99.014	98.747	110.109	107.101	104.094	103.024	102.356	113.718	94.804	96.274	100.819	114.453	76.558	99.749	110.911	98.814	96.274	89.858	107.636	112.515	89.591	84.177	90.393	96.007	22.155	72.481	96.140	79.432	84.378	18.881	50.292	71.011	31.846	41.404	50.025	40.802	0.635	13.935	33.651	34.987
AVE	100.000				103.743				105.798				101.587				96.508				101.571				90.042				67.552				56.140				41.019				20.802			
SD	2.498				5.748				5.328				8.951				14.390				10.357				4.841				31.852				28.525				7.426				16.535			
SEM	1.249				2.874				2.664				4.475				7.195				5.178				2.420				15.926				14.262				3.713				8.267			

BstrR cycle 7 (——)

Dose (µg/mL)	0.000	0.000	0.000	0.000	1.000	1.000	1.000	1.000	2.000	2.000	2.000	2.000	4.000	4.000	4.000	4.000	8.000	8.000	8.000	8.000	16.000	16.000	16.000	16.000	32.000	32.000	32.000	32.000	64.000	64.000	64.000	64.000	128.000	128.000	128.000	128.000	256.000	256.000	256.000	256.000	512.000	512.000	512.000	512.000
% bacteria growth	102.092	97.084	99.061	101.763	107.891	103.937	100.379	105.717	98.138	95.832	98.929	104.794	100.511	93.657	97.809	101.829	88.715	84.300	91.812	97.611	82.521	88.122	88.583	93.855	69.209	69.012	108.089	105.519	91.614	101.565	111.582	111.647	76.919	103.608	106.507	96.030	91.483	82.389	99.918	103.674	63.410	66.969	62.685	51.614
AVE	100.000				104.481				99.423				98.451				90.610				88.270				87.957				104.102				95.766				94.366				61.170			
SD	2.371				3.177				3.814				3.608				5.594				4.632				21.788				9.579				13.318				9.473				6.639			
SEM	1.186				1.588				1.907				1.804				2.797				2.316				10.894				4.789				6.659				4.737				3.320			

Table S2. Dose response curve data

DOXYCYCLINE

*Only the raw data for the cycles represented in Figure 2 are shown in this table

WT cycle 2 (------)

Dose (µg/mL)	0.000	0.000	0.000	0.000	1.000	1.000	1.000	1.000	2.000	2.000	2.000	2.000	4.000	4.000	4.000	4.000	8.000	8.000	8.000	8.000	#####	#####	#####	#####	#####	#####	#####	#####	#####	#####	#####	#####	######	######	######	######	######	######	######	######	######	######	######	######
% bacteria growth	######	99.423	99.361	######	97.303	96.679	98.862	97.428	84.518	83.333	83.396	84.269	#####	67.867	29.950	51.466	#####	#####	#####	8.871	6.751	#####	8.060	5.940	5.129	8.559	6.252	5.878	5.192	5.379	4.880	4.069	3.882	4.693	4.256	4.381	4.506	5.005	4.755	4.506	9.370	13.237	10.056	13.860
AVE	100.000				97.568				83.879				48.394				14.577				7.827				6.455				4.880				4.303				4.693				11.631			
SD	0.731				0.923				0.603				15.765				4.589				2.018				1.479				0.578				0.335				0.239				2.246			
SEM	0.366				0.461				0.302				7.883				2.295				1.009				0.739				0.289				0.168				0.119				1.123			

WT cycle 3 (------)

Dose (µg/mL)	0.000	0.000	0.000	0.000	1.000	1.000	1.000	1.000	2.000	2.000	2.000	2.000	4.000	4.000	4.000	4.000	8.000	8.000	8.000	8.000	#####	#####	#####	#####	#####	#####	#####	#####	#####	#####	#####	#####	######	######	######	######	######	######	######	######	######	######	######	######
% bacteria growth	######	99.699	99.446	######	99.636	######	97.864	98.876	######	98.750	######	97.800	#####	93.116	95.775	95.078	#####	#####	#####	#####	#####	#####	8.989	#####	#####	8.989	7.913	#####	#####	4.558	4.051	#####	5.824	4.241	3.545	8.799	5.254	4.937	4.494	8.419	9.052	6.267	5.950	10.318
AVE	100.000				99.225				100.443				94.873				62.937				20.763				12.455				8.324				5.602				5.776				7.897			
SD	0.606				1.129				2.535				1.206				15.607				11.995				4.654				4.664				2.335				1.789				2.133			
SEM	0.303				0.565				1.268				0.603				7.803				5.998				2.327				2.332				1.167				0.895				1.066			

WT cycle 5 (------)

Dose (µg/mL)	0.000	0.000	0.000	0.000	1.000	1.000	1.000	1.000	2.000	2.000	2.000	2.000	4.000	4.000	4.000	4.000	8.000	8.000	8.000	8.000	#####	#####	#####	#####	#####	#####	#####	#####	#####	#####	#####	#####	######	######	######	######	######	######	######	######	######	######	######	######
% bacteria growth	######	99.888	######	99.373	99.952	######	######	99.502	99.759	######	######	######	#####	######	97.960	94.618	#####	#####	#####	#####	#####	#####	#####	#####	#####	#####	#####	#####	#####	#####	#####	#####	13.576	6.700	4.708	6.057	12.034	7.214	2.715	4.193	12.163	19.875	16.404	19.103
AVE	100.000				102.089				100.771				96.851				73.233				46.385				24.438				20.148				7.760				6.539				16.886			
SD	0.482				3.293				0.927				3.050				12.511				14.968				7.701				9.965				3.965				4.114				3.483			
SEM	0.241				1.647				0.464				1.525				6.256				7.484				3.850				4.983				1.983				2.057				1.741			

WT cycle 10 (------)

Dose (µg/mL)	0.000	0.000	0.000	0.000	1.000	1.000	1.000	1.000	2.000	2.000	2.000	2.000	4.000	4.000	4.000	4.000	8.000	8.000	8.000	8.000	#####	#####	#####	#####	#####	#####	#####	#####	#####	#####	#####	#####	######	######	######	######	######	######	######	######	######	######	######	######
% bacteria growth	95.711	94.784	######	######	######	97.566	######	######	99.884	96.639	######	######	#####	90.677	99.752	######	#####	#####	#####	#####	#####	#####	#####	#####	8.213	#####	#####	#####	0.861	7.286	#####	#####	7.683	5.299	18.480	26.759	-0.530	6.027	19.275	27.819	2.848	10.664	22.322	28.680
AVE	100.000				103.378				102.186				97.715				91.936				60.275				18.712				13.082				14.555				13.148				16.128			
SD	5.758				5.694				5.919				6.460				3.898				6.139				10.056				11.681				9.954				12.788				11.578			
SEM	2.879				2.847				2.959				3.230				1.949				3.070				5.028				5.841				4.977				6.394				5.789			

BstrR cycle 2 (——)

Dose (µg/mL)	0.000	0.000	0.000	0.000	1.000	1.000	1.000	1.000	2.000	2.000	2.000	2.000	4.000	4.000	4.000	4.000	8.000	8.000	8.000	8.000	#####	#####	#####	#####	#####	#####	#####	#####	#####	#####	#####	#####	######	######	######	######	######	######	######	######	######	######	######	######
% bacteria growth	99.387	######	######	######	96.871	95.173	94.167	95.550	70.267	82.783	83.349	78.695	#####	42.909	31.148	41.399	#####	#####	#####	#####	9.198	7.248	5.613	#####	#####	4.418	1.903	4.670	5.613	3.160	0.582	3.160	2.406	1.525	0.204	3.664	2.531	2.154	0.833	4.607	4.041	5.110	6.179	13.097
AVE	100.000				95.440				78.774				35.016				13.616				8.066				5.393				3.129				1.950				2.531				7.107			
SD	0.422				1.118				6.038				8.686				3.887				2.044				3.678				2.054				1.457				1.563				4.088			
SEM	0.211				0.559				3.019				4.343				1.943				1.022				1.839				1.027				0.729				0.782				2.044			

BstrR cycle 5 (——)

Dose (µg/mL)	0.000	0.000	0.000	0.000	1.000	1.000	1.000	1.000	2.000	2.000	2.000	2.000	4.000	4.000	4.000	4.000	8.000	8.000	8.000	8.000	#####	#####	#####	#####	#####	#####	#####	#####	#####	#####	#####	#####	######	######	######	######	######	######	######	######	######	######	######	######
% bacteria growth	97.792	93.086	99.210	######	94.247	88.187	95.536	######	84.384	85.479	90.250	######	#####	80.000	66.978	53.054	#####	#####	#####	#####	6.640	2.965	#####	#####	1.160	1.869	#####	#####	-0.580	3.739	#####	#####	-1.289	2.579	19.597	29.073	0.451	5.093	21.982	29.976	-1.289	8.122	26.172	32.168
AVE	100.000				95.907				90.314				73.425				41.708				14.247				11.974				12.683				12.490				14.376				16.293			
SD	7.107				7.247				7.656				17.412				29.263				11.953				13.035				13.660				14.302				13.920				15.551			
SEM	3.554				3.623				3.828				8.706				14.631				5.976				6.517				6.830				7.151				6.960				7.775			

BstrR cycle 6 (——)

Dose (µg/mL)	0.000	0.000	0.000	0.000	1.000	1.000	1.000	1.000	2.000	2.000	2.000	2.000	4.000	4.000	4.000	4.000	8.000	8.000	8.000	8.000	#####	#####	#####	#####	#####	#####	#####	#####	#####	#####	#####	#####	######	######	######	######	######	######	######	######	######	######	######	######
% bacteria growth	######	99.599	99.984	######	99.663	######	99.085	######	98.314	98.443	97.415	######	#####	98.250	97.736	99.534	#####	#####	#####	#####	#####	#####	#####	#####	#####	#####	#####	#####	#####	7.770	#####	#####	6.678	5.779	15.476	17.916	10.275	3.853	12.329	17.916	17.852	10.724	19.072	25.558
AVE	100.000				100.128				99.117				98.507				87.831				88.939				58.372				21.689				11.463				11.093				18.301			
SD	0.289				1.131				2.168				0.756				5.681				2.229				13.155				15.024				6.136				5.807				6.079			
SEM	0.144				0.566				1.084				0.378				2.840				1.115				6.578				7.512				3.068				2.904				3.040			

BstrR cycle 7 (——)

Dose (µg/mL)	0.000	0.000	0.000	0.000	1.000	1.000	1.000	1.000	2.000	2.000	2.000	2.000	4.000	4.000	4.000	4.000	8.000	8.000	8.000	8.000	#####	#####	#####	#####	#####	#####	#####	#####	#####	#####	#####	#####	######	######	######	######	######	######	######	######	######	######	######	######
% bacteria growth	99.087	97.890	98.331	######	96.190	99.213	99.402	######	######	97.639	99.087	######	#####	96.820	######	######	#####	#####	#####	#####	#####	#####	#####	#####	#####	#####	#####	#####	#####	#####	#####	#####	21.568	21.820	31.014	32.651	20.120	22.009	25.472	28.936	14.893	14.767	23.520	27.802
AVE	100.000				99.355				100.866				100.520				87.437				91.562				58.674				32.651				26.763				24.134				20.246			
SD	3.166				2.624				3.982				4.169				9.072				3.607				16.891				2.364				5.892				3.894				6.493			
SEM	1.583				1.312				1.991				2.084				4.536				1.804				8.446				1.182				2.946				1.947				3.247			

Table S2. Dose response curve data

AMOXICILLIN

*Only the raw data for the cycles represented in Figure S1 are shown in this table

WT cycle 3 (------)

Dose (μg/mL)	0.000	0.000	0.000	0.000	0.100	0.100	0.100	0.100	0.200	0.200	0.200	0.200	0.400	0.400	0.400	0.400	0.800	0.800	0.800	0.800	1.600	1.600	1.600	1.600	3.200	3.200	3.200	3.200	6.400	6.400	6.400	6.400	12.800	12.800	12.800	12.800	25.600	25.600	25.600	25.600	51.200	51.200	51.200	51.200
% bacteria growth	101.462	99.196	100.146	99.196	90.132	92.544	95.833	94.737	74.561	75.585	66.594	81.725	15.497	12.354	11.988	12.573	5.336	3.728	4.020	3.143	3.582	3.582	3.728	3.874	4.532	4.605	3.655	2.705	1.389	1.462	1.535	1.316	1.023	1.170	1.170	1.170	0.804	0.585	1.096	1.096	0.073	1.535	-0.585	1.389
AVE		100.000				93.311				74.616				13.103				4.057				3.692				3.874				1.425				1.133				0.895				0.603		
SD		1.073				2.523				6.214				1.614				0.928				0.140				0.891				0.094				0.073				0.249				1.029		
SEM		0.536				1.261				3.107				0.807				0.464				0.070				0.446				0.047				0.037				0.124				0.515		

WT cycle 4 (------)

Dose (μg/mL)	0.000	0.000	0.000	0.000	0.100	0.100	0.100	0.100	0.200	0.200	0.200	0.200	0.400	0.400	0.400	0.400	0.800	0.800	0.800	0.800	1.600	1.600	1.600	1.600	3.200	3.200	3.200	3.200	6.400	6.400	6.400	6.400	12.800	12.800	12.800	12.800	25.600	25.600	25.600	25.600	51.200	51.200	51.200	51.200
% bacteria growth	98.825	100.783	103.760	96.632	104.621	102.115	109.790	107.519	90.131	90.601	96.710	103.290	81.281	83.474	87.625	86.998	59.428	58.488	21.128	23.478	12.277	17.055	20.658	8.361	5.541	6.481	6.403	7.734	6.951	5.541	6.325	7.500	3.348	3.348	3.975	3.975	2.800	2.800	3.583	4.367	2.643	2.957	2.252	1.860
AVE		100.000				106.011				95.183				84.844				40.631				14.588				6.540				6.579				3.662				3.388				2.428		
SD		3.026				3.350				6.180				2.997				21.188				5.387				0.903				0.842				0.362				0.750				0.476		
SEM		1.513				1.675				3.090				1.499				10.594				2.693				0.451				0.421				0.181				0.375				0.238		

WT cycle 7 (------)

Dose (μg/mL)	0.000	0.000	0.000	0.000	0.100	0.100	0.100	0.100	0.200	0.200	0.200	0.200	0.400	0.400	0.400	0.400	0.800	0.800	0.800	0.800	1.600	1.600	1.600	1.600	3.200	3.200	3.200	3.200	6.400	6.400	6.400	6.400	12.800	12.800	12.800	12.800	25.600	25.600	25.600	25.600	51.200	51.200	51.200	51.200
% bacteria growth	100.838	103.695	101.035	94.432	105.666	105.962	112.072	109.805	102.217	109.904	102.119	116.112	110.988	109.608	111.284	112.959	115.718	109.411	99.852	113.944	106.652	107.440	100.345	103.794	92.363	89.308	83.493	96.206	59.547	42.695	45.060	25.055	2.291	3.375	1.798	2.094	1.503	1.404	1.306	0.912	1.109	1.700	0.517	1.503
AVE		100.000				108.376				107.588				111.210				109.731				104.558				90.342				43.089				2.390				1.281				1.207		
SD		3.934				3.102				6.752				1.376				7.101				3.216				5.368				14.143				0.687				0.259				0.521		
SEM		1.967				1.551				3.376				0.688				3.551				1.608				2.684				7.072				0.344				0.130				0.261		

WT cycle 10 (------)

Dose (μg/mL)	0.000	0.000	0.000	0.000	0.100	0.100	0.100	0.100	0.200	0.200	0.200	0.200	0.400	0.400	0.400	0.400	0.800	0.800	0.800	0.800	1.600	1.600	1.600	1.600	3.200	3.200	3.200	3.200	6.400	6.400	6.400	6.400	12.800	12.800	12.800	12.800	25.600	25.600	25.600	25.600	51.200	51.200	51.200	51.200
% bacteria growth	96.400	98.866	101.924	102.811	101.726	105.475	112.577	120.370	100.937	108.434	112.873	122.145	97.781	117.016	105.179	110.703	106.856	114.254	124.414	112.873	89.100	130.826	91.665	136.843	78.644	83.773	85.943	97.879	87.916	78.052	77.855	86.141	31.492	32.478	44.809	45.401	2.096	-1.850	7.226	12.256	1.504	-0.271	8.409	11.862
AVE		100.000				110.037				111.097				107.670				114.599				112.109				86.560				82.491				38.545				4.932				5.376		
SD		2.936				8.228				8.861				8.176				7.289				25.229				8.143				5.290				7.589				6.136				5.720		
SEM		1.468				4.114				4.430				4.088				3.645				12.615				4.072				2.645				3.795				3.068				2.860		

BstrR cycle 1 (——)

Dose (μg/mL)	0.000	0.000	0.000	0.000	0.100	0.100	0.100	0.100	0.200	0.200	0.200	0.200	0.400	0.400	0.400	0.400	0.800	0.800	0.800	0.800	1.600	1.600	1.600	1.600	3.200	3.200	3.200	3.200	6.400	6.400	6.400	6.400	12.800	12.800	12.800	12.800	25.600	25.600	25.600	25.600	51.200	51.200	51.200	51.200
% bacteria growth	100.697	99.984	100.632	98.687	108.156	103.421	104.070	107.832	76.180	71.639	53.413	56.008	16.377	22.604	18.258	13.459	16.637	17.804	12.940	5.092	13.718	9.372	10.021	2.951	9.956	8.854	6.648	6.389	9.308	8.659	5.156	4.119	10.929	7.362	-0.292	2.627	6.324	3.146	2.173	1.849	9.048	5.675	2.757	7.816
AVE		100.000				105.870				64.310				17.675				13.118				9.016				7.962				6.810				5.156				3.373				6.324		
SD		0.933				2.471				11.288				3.834				5.739				4.473				1.729				2.558				4.976				2.043				2.756		
SEM		0.467				1.235				5.644				1.917				2.869				2.237				0.865				1.279				2.488				1.022				1.378		

BstrR cycle 2 (——)

Dose (μg/mL)	0.000	0.000	0.000	0.000	0.100	0.100	0.100	0.100	0.200	0.200	0.200	0.200	0.400	0.400	0.400	0.400	0.800	0.800	0.800	0.800	1.600	1.600	1.600	1.600	3.200	3.200	3.200	3.200	6.400	6.400	6.400	6.400	12.800	12.800	12.800	12.800	25.600	25.600	25.600	25.600	51.200	51.200	51.200	51.200
% bacteria growth	96.185	93.014	102.408	108.392	80.389	29.289	63.515	94.690	30.127	16.126	28.990	71.414	6.193	13.313	25.221	27.794	1.526	7.210	21.331	27.016	6.372	10.621	21.810	28.990	6.791	32.700	20.553	31.204	8.586	13.014	25.580	31.982	8.227	15.946	25.639	30.726	5.894	14.390	26.417	32.281	11.399	14.749	28.512	28.691
AVE		100.000				66.971				36.664				18.130				14.271				16.948				22.812				19.791				20.135				19.746				20.838		
SD		6.821				28.167				24.021				10.155				11.896				10.337				11.972				10.857				10.030				11.863				9.069		
SEM		3.410				14.084				12.010				5.077				5.948				5.168				5.986				5.428				5.015				5.932				4.534		

BstrR cycle 3 (——)

Dose (μg/mL)	0.000	0.000	0.000	0.000	0.100	0.100	0.100	0.100	0.200	0.200	0.200	0.200	0.400	0.400	0.400	0.400	0.800	0.800	0.800	0.800	1.600	1.600	1.600	1.600	3.200	3.200	3.200	3.200	6.400	6.400	6.400	6.400	12.800	12.800	12.800	12.800	25.600	25.600	25.600	25.600	51.200	51.200	51.200	51.200
% bacteria growth	98.966	96.997	99.951	104.087	102.577	97.916	106.187	102.511	5.416	1.477	96.668	51.830	0.689	9.421	38.831	37.912	3.053	4.037	19.465	31.413	1.280	6.138	20.187	32.595	-0.952	6.073	22.025	32.398	2.921	7.976	23.995	33.973	1.149	8.633	25.964	34.302	-0.361	10.340	25.964	35.155	0.624	11.718	26.489	32.989
AVE		100.000				102.298				38.848				21.713				14.492				15.050				14.886				17.216				17.512				17.774				17.955		
SD		2.988				3.389				44.818				19.567				13.555				14.180				15.123				14.335				15.274				15.846				14.584		
SEM		1.494				1.694				22.409				9.783				6.777				7.090				7.562				7.167				7.637				7.923				7.292		

BstrR cycle 4 (——)

Dose (μg/mL)	0.000	0.000	0.000	0.000	0.100	0.100	0.100	0.100	0.200	0.200	0.200	0.200	0.400	0.400	0.400	0.400	0.800	0.800	0.800	0.800	1.600	1.600	1.600	1.600	3.200	3.200	3.200	3.200	6.400	6.400	6.400	6.400	12.800	12.800	12.800	12.800	25.600	25.600	25.600	25.600	51.200	51.200	51.200	51.200
% bacteria growth	97.987	109.217	97.987	94.808	97.582	110.367	105.226	97.920	93.387	93.387	103.602	99.882	103.061	103.602	99.814	93.049	78.167	102.385	87.367	98.461	100.152	95.079	82.496	72.620	54.017	79.114	57.737	66.125	76.408	80.467	102.655	89.734	82.090	96.161	105.361	100.288	84.526	87.705	89.870	91.493	72.078	56.858	76.814	58.143
AVE		100.000				102.774				97.565				99.882				91.595				87.587				64.248				87.316				95.975				88.398				65.973		
SD		6.325				6.170				5.057				4.852				10.981				12.436				11.129				11.648				9.992				3.012				9.987		
SEM		3.162				3.085				2.528				2.426				5.491				6.218				5.565				5.824				4.996				1.506				4.993		

Table S2. Dose response curve data

PIPERACILLIN

*Only the raw data for the cycles represented in Figure S1 are shown in this table

WT cycle 2 (------)

Dose (µg/mL)	0.000	0.000	0.000	0.000	0.100	0.100	0.100	0.100	0.200	0.200	0.200	0.200	0.400	0.400	0.400	0.400	0.800	0.800	0.800	0.800	1.600	1.600	1.600	1.600	3.200	3.200	3.200	3.200	6.400	6.400	6.400	6.400	12.800	12.800	12.800	12.800	25.600	25.600	25.600	25.600	51.200	51.200	51.200	51.200
% bacteria growth	98.024	101.275	102.996	97.642	88.209	96.622	96.048	82.218	61.058	71.256	68.069	60.038	16.316	11.727	16.571	11.727	13.894	10.134	8.987	6.182	4.971	5.864	1.402	4.780	6.119	3.187	0.382	5.417	7.521	1.083	1.020	1.976	5.609	2.486	2.613	3.569	7.393	2.549	2.804	1.083	2.294	5.035	-0.637	2.741
AVE	99.984				90.774				65.105				14.085				9.799				4.254				3.776				2.900				3.569				3.458				2.358			
SD	2.586				6.875				5.436				2.725				3.195				1.959				2.585				3.111				1.443				2.731				2.330			
SEM	1.293				3.438				2.718				1.362				1.597				0.980				1.292				1.556				0.722				1.366				1.165			

WT cycle 3 (------)

Dose (µg/mL)	0.000	0.000	0.000	0.000	0.100	0.100	0.100	0.100	0.200	0.200	0.200	0.200	0.400	0.400	0.400	0.400	0.800	0.800	0.800	0.800	1.600	1.600	1.600	1.600	3.200	3.200	3.200	3.200	6.400	6.400	6.400	6.400	12.800	12.800	12.800	12.800	25.600	25.600	25.600	25.600	51.200	51.200	51.200	51.200
% bacteria growth	90.268	101.678	101.946	106.040	95.705	103.423	97.718	102.013	87.383	91.477	89.732	83.154	63.087	60.671	60.738	55.772	10.067	7.315	6.510	6.174	5.973	-1.544	3.154	0.537	0.470	0.671	4.832	1.208	3.020	2.483	1.745	2.886	3.356	-4.228	3.154	2.349	1.409	4.430	7.450	6.510	3.490	1.477	4.765	0.403
AVE	99.983				99.715				87.936				60.067				7.517				2.030				1.795				2.534				1.158				4.950				2.534			
SD	6.777				3.610				3.602				3.076				1.766				3.256				2.048				0.573				3.617				2.676				1.962			
SEM	3.389				1.805				1.801				1.538				0.883				1.628				1.024				0.287				1.808				1.338				0.981			

WT cycle 7 (------)

Dose (µg/mL)	0.000	0.000	0.000	0.000	0.100	0.100	0.100	0.100	0.200	0.200	0.200	0.200	0.400	0.400	0.400	0.400	0.800	0.800	0.800	0.800	1.600	1.600	1.600	1.600	3.200	3.200	3.200	3.200	6.400	6.400	6.400	6.400	12.800	12.800	12.800	12.800	25.600	25.600	25.600	25.600	51.200	51.200	51.200	51.200
% bacteria growth	100.961	100.577	97.566	100.769	96.477	99.167	96.925	98.591	97.309	99.423	93.017	98.591	92.184	92.184	90.583	94.170	35.746	34.081	35.939	31.967	7.687	7.751	2.883	18.962	3.780	1.858	3.587	13.773	3.972	-0.577	10.250	15.119	1.794	-0.128	14.862	11.851	2.627	1.537	12.684	13.709	2.562	9.994	14.862	10.378
AVE	99.968				97.790				97.085				92.281				34.433				9.321				5.750				7.191				7.095				7.639				9.449			
SD	1.609				1.292				2.848				1.469				1.844				6.820				5.419				6.902				7.376				6.446				5.095			
SEM	0.805				0.646				1.424				0.734				0.922				3.410				2.709				3.451				3.688				3.223				2.548			

WT cycle 9 (------)

Dose (µg/mL)	0.000	0.000	0.000	0.000	0.100	0.100	0.100	0.100	0.200	0.200	0.200	0.200	0.400	0.400	0.400	0.400	0.800	0.800	0.800	0.800	1.600	1.600	1.600	1.600	3.200	3.200	3.200	3.200	6.400	6.400	6.400	6.400	12.800	12.800	12.800	12.800	25.600	25.600	25.600	25.600	51.200	51.200	51.200	51.200
% bacteria growth	99.348	100.237	100.296	100.118	100.592	100.770	102.310	97.927	98.756	99.585	100.533	99.348	94.491	94.964	95.320	89.573	70.142	76.422	68.365	61.256	16.588	20.024	19.313	19.491	6.043	5.450	9.716	7.109	4.976	9.834	3.910	6.991	6.457	5.213	4.265	11.137	9.301	5.806	7.701	6.339	3.377	4.502	11.552	6.991
AVE	100.000				100.400				99.556				93.587				69.046				18.854				7.079				6.428				6.768				7.287				6.605			
SD	0.441				1.820				0.739				2.697				6.238				1.541				1.887				2.606				3.048				1.562				3.627			
SEM	0.220				0.910				0.370				1.349				3.119				0.770				0.943				1.303				1.524				0.781				1.813			

BstrR cycle 2 (——)

Dose (µg/mL)	0.000	0.000	0.000	0.000	0.100	0.100	0.100	0.100	0.200	0.200	0.200	0.200	0.400	0.400	0.400	0.400	0.800	0.800	0.800	0.800	1.600	1.600	1.600	1.600	3.200	3.200	3.200	3.200	6.400	6.400	6.400	6.400	12.800	12.800	12.800	12.800	25.600	25.600	25.600	25.600	51.200	51.200	51.200	51.200
% bacteria growth	101.601	96.570	99.249	102.581	95.263	97.419	92.649	97.288	81.542	81.085	90.363	85.854	22.476	23.587	22.803	21.692	11.434	19.013	11.303	12.610	9.082	8.690	7.579	8.363	8.298	3.855	5.031	8.037	6.338	7.775	4.378	5.031	4.312	3.920	3.398	4.051	3.202	4.966	4.704	4.835	2.614	3.724	2.940	5.423
AVE	100.000				95.655				84.711				22.640				13.590				8.429				6.305				5.880				3.920				4.427				3.675			
SD	2.680				2.234				4.337				0.785				3.663				0.638				2.206				1.503				0.385				0.824				1.255			
SEM	1.340				1.117				2.169				0.392				1.831				0.319				1.103				0.752				0.192				0.412				0.627			

BstrR cycle 3 (——)

Dose (µg/mL)	0.000	0.000	0.000	0.000	0.100	0.100	0.100	0.100	0.200	0.200	0.200	0.200	0.400	0.400	0.400	0.400	0.800	0.800	0.800	0.800	1.600	1.600	1.600	1.600	3.200	3.200	3.200	3.200	6.400	6.400	6.400	6.400	12.800	12.800	12.800	12.800	25.600	25.600	25.600	25.600	51.200	51.200	51.200	51.200
% bacteria growth	101.931	98.827	100.776	98.466	92.184	104.892	102.653	87.563	83.664	94.278	92.834	91.390	61.859	66.841	63.520	63.736	12.401	8.285	12.906	9.657	5.758	5.181	5.397	5.542	5.542	4.747	2.798	3.159	3.953	3.881	3.014	4.170	3.087	4.242	3.159	2.509	3.736	3.736	2.870	3.014	2.509	2.437	3.087	2.581
AVE	100.000				96.823				90.542				63.989				10.812				5.469				4.061				3.755				3.249				3.339				2.653			
SD	1.639				8.294				4.734				2.078				2.208				0.243				1.300				0.508				0.723				0.462				0.295			
SEM	0.820				4.147				2.367				1.039				1.104				0.122				0.650				0.254				0.361				0.231				0.147			

BstrR cycle 6 (——)

Dose (µg/mL)	0.000	0.000	0.000	0.000	0.100	0.100	0.100	0.100	0.200	0.200	0.200	0.200	0.400	0.400	0.400	0.400	0.800	0.800	0.800	0.800	1.600	1.600	1.600	1.600	3.200	3.200	3.200	3.200	6.400	6.400	6.400	6.400	12.800	12.800	12.800	12.800	25.600	25.600	25.600	25.600	51.200	51.200	51.200	51.200
% bacteria growth	100.493	101.703	99.220	98.583	97.055	96.418	102.085	103.168	97.564	94.826	96.928	94.126	74.769	84.830	74.578	82.728	34.082	37.775	38.220	31.598	13.451	16.507	15.234	15.298	6.702	6.765	4.982	8.803	5.301	4.218	3.518	5.938	5.492	4.537	1.608	3.009	4.091	5.365	3.773	3.836	1.926	4.027	2.690	2.181
AVE	100.000				99.682				95.861				79.226				35.419				15.123				6.813				4.744				3.661				4.266				2.706			
SD	1.386				3.439				1.645				5.327				3.151				1.259				1.563				1.082				1.709				0.745				0.936			
SEM	0.693				1.719				0.823				2.664				1.575				0.629				0.781				0.541				0.854				0.373				0.468			

BstrR cycle 7 (——)

Dose (µg/mL)	0.000	0.000	0.000	0.000	0.100	0.100	0.100	0.100	0.200	0.200	0.200	0.200	0.400	0.400	0.400	0.400	0.800	0.800	0.800	0.800	1.600	1.600	1.600	1.600	3.200	3.200	3.200	3.200	6.400	6.400	6.400	6.400	12.800	12.800	12.800	12.800	25.600	25.600	25.600	25.600	51.200	51.200	51.200	51.200
% bacteria growth	100.693	98.551	100.630	100.126	97.795	101.512	102.079	95.654	96.913	96.031	98.299	97.417	72.976	82.740	81.165	76.063	13.512	20.441	15.780	18.803	5.575	6.268	3.244	5.071	5.953	3.307	2.362	4.000	4.693	1.921	0.220	3.370	3.433	1.732	0.913	3.937	1.795	0.850	0.409	3.748	1.669	1.921	0.283	3.811
AVE	100.000				99.260				97.165				78.236				17.134				5.039				3.906				2.551				2.504				1.701				1.921			
SD	0.999				3.064				0.948				4.519				3.092				1.294				1.521				1.922				1.419				1.482				1.451			
SEM	0.499				1.532				0.474				2.259				1.546				0.647				0.760				0.961				0.710				0.741				0.726			

Table S3 The identities of the transconjugants, their coverage and similarity percentages as determined from the NCBI BLAST tool.

Project Number	Sample Number	Bacteria	Description	Max Score	Total Score	Query Cover	E value	Per. Ident.	Accession
S8139-1	HCTBE2B	*Enterococcus faecalis*	Enterococcus faecalis strain SCAID PHRX1-2018 chromosome	2355	1275	100	0	100	CP041877.1
S8139-2	HCTBE2C	*Enterococcus faecalis*	Enterococcus faecalis strain SCAID PHRX1-2018 chromosome	2355	1275	100	0	100	CP041877.1
S8139-3	HCTBE3B	*Enterococcus faecalis*	Enterococcus faecalis strain SCAID PHRX1-2018 chromosome	2355	1275	100	0	100	CP041877.1
S8139-5	CacoBE3B	*Enterococcus faecalis*	Enterococcus faecalis strain pk13 16S ribosomal RNA gene, partial sequence	2763	2763	100	0	100	PP886470.1
S8139-6	CacoBE3C	*Enterococcus faecalis*	Enterococcus faecalis strain SCAID PHRX1-2018 chromosome	2355	1275	100	0	100	CP041877.1
